# A systematic review and meta-analysis of neuromodulation therapies for substance use disorders

**DOI:** 10.1038/s41386-023-01776-0

**Published:** 2023-12-12

**Authors:** Dhvani D. Mehta, Angela Praecht, Heather B. Ward, Marcos Sanches, Maryam Sorkhou, Victor M. Tang, Vaughn R. Steele, Colleen A. Hanlon, Tony P. George

**Affiliations:** 1 Addictions Division, CAMH, Toronto, ON Canada; 2 Department of Psychiatry, Temerty Faculty of Medicine, University of Toronto, Toronto, ON Canada; 3 Department of Psychiatry and Behavioral Sciences, Vanderbilt University Medical Center, Nashville, TN USA; 4 Department of Psychiatry, Yale University School of Medicine, New Haven, CT USA; 5 Brainsway, Inc., Winston-Salem, NC USA

**Keywords:** Outcomes research, Diseases

## Abstract

While pharmacological, behavioral and psychosocial treatments are available for substance use disorders (SUDs), they are not always effective or well-tolerated. Neuromodulation (NM) methods, including repetitive transcranial magnetic stimulation (rTMS), transcranial direct current stimulation (tDCS) and deep brain stimulation (DBS) may address SUDs by targeting addiction neurocircuitry. We evaluated the efficacy of NM to improve behavioral outcomes in SUDs. A systematic literature search was performed on MEDLINE, PsychINFO, and PubMed databases and a list of search terms for four key concepts (SUD, rTMS, tDCS, DBS) was applied. Ninety-four studies were identified that examined the effects of rTMS, tDCS, and DBS on substance use outcomes (e.g., craving, consumption, and relapse) amongst individuals with SUDs including alcohol, tobacco, cannabis, stimulants, and opioids. Meta-analyses were performed for alcohol and tobacco studies using rTMS and tDCS. We found that rTMS reduced substance use and craving, as indicated by medium to large effect sizes (Hedge’s *g* > 0.5). Results were most encouraging when multiple stimulation sessions were applied, and the left dorsolateral prefrontal cortex (DLPFC) was targeted. tDCS also produced medium effect sizes for drug use and craving, though they were highly variable and less robust than rTMS; right anodal DLPFC stimulation appeared to be most efficacious. DBS studies were typically small, uncontrolled studies, but showed promise in reducing misuse of multiple substances. NM may be promising for the treatment of SUDs. Future studies should determine underlying neural mechanisms of NM, and further evaluate extended treatment durations, accelerated administration protocols and long-term outcomes with biochemical verification of substance use.

## Introduction

Substance use disorders (SUDs) account for 500,000 deaths annually in the U.S alone [1, 2]. Moreover, SUDs frequently co-occur with psychiatric disorders, including schizophrenia and mood disorders [3–5]. Although there are validated pharmacologic and psychotherapeutic treatments available for SUDs, relapse rates are high [6, 7]. Thus, development of neuroscience-informed therapeutics for SUDs is critical. Neuromodulation (NM) may offer such opportunities [8, 9].

Reinforcing effects of substances are primarily mediated by mesocorticolimbic systems, which include midbrain dopamine (DA) projections to prefrontal cortex (PFC) and ventral striatum [nucleus accumbens (NAc)] [10, 11]. Substance misuse is associated with mesolimbic hypodopaminergia [12], and dysfunction of dorsolateral prefrontal cortex (DLPFC) and dorsal anterior cingulate cortex (dACC), which are involved in decision-making and self-control. Moreover, the ventral PFC, including the orbitofrontal cortex (OFC) and ventral anterior cingulate cortex (vACC), is involved in limbic arousal and emotional processing [13]. Hence, dysfunction in these systems has been associated with SUDs [14]. Furthermore, left DLPFC mediates reward-based motivation, while right DLPFC is involved in withdrawal-related behaviors and inhibition [15]. Thus, use of NM to stimulate right DLPFC may strengthen executive functions by inhibiting the left DLPFC to counterbalance hemispheric imbalance, which may contribute to reduction of substance consumption and craving [16, 17]. Invasive and/or non-invasive NM may be promising brain-based approaches since they modulate SUD-related mesolimbocortical circuitry [8, 9, 18]. Such interventions include repetitive transcranial magnetic stimulation (rTMS), transcranial direct current stimulation (tDCS), and deep brain stimulation (DBS).

### Repetitive transcranial magnetic stimulation (rTMS)

**Fig. 1:**
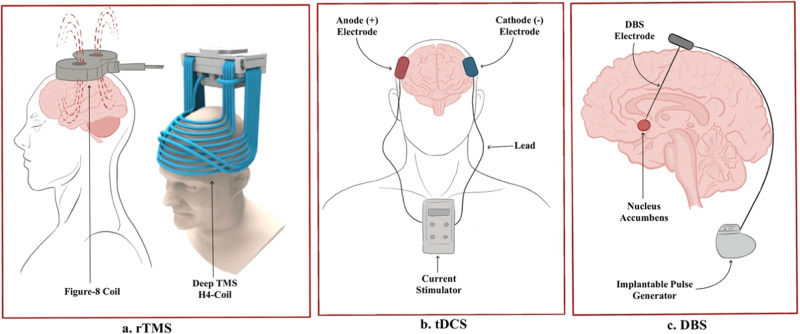
Neuromodulation techniques. Diagrams to illustrate the three neuromodulation techniques investigated: (**a**) rTMS (Deep TMS image acquired from Brainsway, Inc.), (**b**) tDCS, (**c**) DBS.

rTMS is a non-invasive NM technique that has shown utility for neurological and psychiatric disorders [19]. Application of alternating magnetic fields to the scalp through a copper wire induces temporary electrical currents and modulates cortical excitability in localized brain tissue [20] (Fig. 1a). Numerous studies have demonstrated enduring functional and structural neuroplastic changes in target regions [21, 22], and increased DA release in the mesolimbic system [23–26].

Stimulation parameters vary significantly with respect to stimulus intensity, frequency and total number of pulses, which can produce differential effects [27]. Typically, low frequency (LF; ≤1 Hz) stimulation produces local inhibitory effects while high frequency (HF; ≥5 Hz) stimulation produces local excitatory effects on motor cortex [28, 29]. rTMS primarily alters motor cortical excitability and inhibition, with indirect effects on craving or motivation. Frequency-dependent rTMS effects on regional brain activity may have implications for clinical therapeutics in neuropsychiatric disorders [30, 31]. Coil type can also modulate effects; while traditional TMS employs a figure-8 coil design and can only reach depths of 0.7 cm, deep TMS, wherein a three-dimensional H-coil helmet design is used, can stimulate a deeper and broader brain area, reaching a depth of 3.2 cm [32].

Two robust rTMS adaptations have emerged wherein bursts of magnetic pulses, referred to as theta burst stimulation (TBS), are applied. In intermittent theta burst stimulation (iTBS), a two second train of TBS bursts is repeated every ten seconds, inducing long-term potentiation and cortical excitability [33, 34]. Contrastingly, continuous theta-burst stimulation (cTBS) applies trains of uninterrupted TBS bursts and induces long-term depression and inhibitory effects [34].

rTMS appears safe when administered according to recommended guidelines [35]. There is little risk beyond local discomfort at the site of stimulation and other minor side effects (e.g. mild headache, dizziness) [36]. Importantly, a deep-TMS system was recently cleared by the Food and Drug Administration (FDA) for smoking cessation [37]. However, long-term effects of repeated rTMS sessions are unknown [38].

### Transcranial direct current stimulation (tDCS)

Using two or more electrodes (i.e., anodal, cathodal), tDCS delivers a low intensity current (0.5–2.0 milliamps [mA]) to a targeted brain region for several minutes (Fig. 1b). This allows for polarity-dependent modulation of the neuronal resting membrane potential and cortical excitability. Cathodal current decreases while anodal current increases cortical excitability [39, 40]. Similar to rTMS, tDCS protocols can vary with respect to numerous parameters such as current strength, electrode size and placement, stimulation duration and frequency [41].

tDCS is an accessible, low-cost stimulation method that is well-tolerated, though minor side effects such as scalp irritation are reported [42]. Similar to rTMS, tDCS has been used to effectively treat neuropsychiatric conditions such as Parkinson’s disease, chronic pain, and major depression [43]. Although underlying mechanisms for tDCS are not fully understood, induction of neurochemical changes in targeted brain tissue is being investigated for SUD treatment.

### Deep brain stimulation (DBS)

DBS is an invasive NM technique used to treat Alzheimer’s disease, Parkinson’s disease, and obsessive compulsive disorder [44]. It involves a neurosurgical procedure wherein implanted electrodes deliver electrical pulses directly to targeted brain regions, which modulates neural circuitry and subsequently alters neuroplasticity (Fig. 1c). While rTMS and tDCS use lower frequencies to induce excitation or inhibition of neurons, DBS blocks neural transmission with high-frequency stimulation [45]. Implanted electrodes are connected to an implantable pulse generator placed under the skin of the chest wall, allowing for continuous stimulation at a pre-set frequency [46]. Thus, stimulation parameters can be modulated as a patient’s condition changes.

Unlike other surgical interventions, DBS does not damage brain tissue [47], but given its invasive nature, is associated with infection, seizures or stroke. DBS is well-tolerated once the patient has recovered from the primary surgical procedure [48]. Focal stimulation of deep brain regions involved in addiction neurocircuitry (e.g. NAc) may facilitate SUD treatment.

We conducted a systematic review and meta-analysis to determine the efficacy of NM for improving addiction outcomes (e.g., drug craving, consumption, and relapse). As significant progress has been made in this area, a systematic review and meta-analysis building on previous narrative reviews [8, 9] with quantification of NM effects in SUDs is warranted.

## Methods

### Search strategy

A comprehensive literature search by two authors (D.M. and A.P., trained on Covidence) was conducted using Medline, PubMed and PsycINFO databases, in accordance with Preferred Reporting Items for Systematic Reviews and Meta-Analyses (PRISMA) guidelines [49] (Supplementary Fig. 1), through October, 2023. Articles published after 2000 in peer-reviewed journals were considered. A list of keywords and search terms for four key concepts (SUD, rTMS, tDCS, DBS) was applied (See Supplementary Table 1 for Search Strategy). Reference lists of relevant reviews were also screened for applicable articles. The review was registered at PROSPERO (CRD42023475165).

### Eligibility criteria

Using PICOS [50], studies were included if they satisfied the following criteria – Population (P): Studies recruiting participants (18+ years of age) diagnosed with SUD/dependence of alcohol, tobacco, cocaine, methamphetamine, opioids, or cannabis, according to standardized criteria (e.g., DSM-IV or DSM-5); Intervention (I): Intervention employing either rTMS, tDCS, or DBS; Comparison (C): Studies including either sham stimulation, a control group receiving no intervention or an active control arm were included. DBS studies were exempted considering the ethical constraints on the use of control groups with invasive brain surgery/stimulation; Outcomes (O): Studies investigating substance-related outcomes (consumption, craving, cue-induced craving, abstinence, relapse) as primary or secondary outcomes of interest using a validated measurement tool (e.g. Obsessive Compulsive Drinking Scale [OCDS]); Study Design (S): Studies employing either a parallel (between-subject) or cross-over (within-subject) randomized controlled trial (RCT). For DBS, case series (*N* ≥ 2) were permitted.

Studies were excluded if: (1) recruited participants without a SUD and/or a standardized criteria for diagnosis (e.g., “heavy drinkers”); (2) lacked a well-defined control group (rTMS and tDCS studies); (3) literature review, meta-analysis, dissertation, abstract, conference presentation or case report.

### Study selection

Two authors (D.M. and A.P.) independently screened titles and abstracts obtained on Covidence to determine eligibility for full-text review, and subsequently reviewed the full text of the screened studies. Disagreements were resolved by consensus, and review with the senior author (T.P.G.).

### Data extraction and risk of bias

For included studies, two authors (D.M. and A.P.) extracted author information, sample size, study design, stimulation parameters, primary substance use outcomes (craving and consumption), and any secondary outcomes. Effect sizes (Hedge’s *g*) of substance use and other outcomes were calculated for each study using post-treatment data of active and control (sham and/or no treatment) groups, respectively (see Tables 1–4). Due to the heterogeneity in follow-up periods across studies, treatment effects were determined using end-of-treatment data, unless otherwise stated. For DBS studies with no control conditions, within-subject (pre-post treatment) effect sizes were calculated.

**Table 1:** Repetitive Transcranial Magnetic Stimulation (rTMS) [Total *N* = 2406; Total Studies = 51].

Citation	Sample Size	Study Design	# of Sessions & Targeted Region	Stimulation Intensity	Stimulation Frequency	Coil Type	Craving Effect Size (Hedge’s *g*) [95% CI]	Consumption Effect Size (Hedge’s *g*) [95% CI]	Other Outcome(s) Effect Size (Hedge’s *g*) [95% CI]	Results
**Alcohol**: Single Active Stimulation Session Total *N* = 149; 5 Studies										
Herremans et al. [56]	*N* = 31	A randomized, prospective, single-blind, sham-controlled study with recently detoxified alcohol-dependent participants	1 Active *OR* 1 Sham Session **Right DLPFC** *(w/ MRI-neuronavigation)*	110%	20 Hz	Figure-8 Coil	**Craving** Active vs. Sham: −0.18 [−0.93–0.56]	**NA**	**NA**	No significant effect on alcohol craving was observed following active rTMS compared to sham stimulation.
Herremans et al. [57]	*N* = 29	A randomized, single-blind, sham-controlled, crossover study with recently detoxified, alcohol-dependent participants	1 Active *AND* 1 Sham Session **Right DLPFC** *(w/ MRI-neuronavigation)*	110%	20 Hz	Figure-8 Coil	**Craving** Active vs. Sham: −0.33 [−0.85–0.19]	**NA**	**Executive Functioning** NA	No significant post-treatment effects of active or sham rTMS were observed on alcohol craving, compared to baseline. No significant difference in executive functioning or alcohol craving was observed following one active rTMS session compared to sham.
Herremans et al. [58]	*N* = 26	An open label, sham-controlled, crossover study with recently detoxified alcohol-dependent participants	1 Active *AND* 1 Sham Session **Right DLPFC** *(w/ MRI-neuronavigation)*	110%	20 Hz	Figure-8 Coil	**Craving** Active vs. Sham: 0 [−0.77–0.77]	**NA**	**NA**	No significant difference in alcohol craving was observed following 1 active rTMS session compared to sham.
Hanlon et al. [59]	*N* = 24	A single-blind, sham-controlled, crossover study with alcohol-dependent participants	1 Active *AND* 1 Sham Session of **cTBS** **Left Frontal Pole**	110%	3-pulse bursts presented at 5 Hz	Figure-8 Coil	**Craving** Active vs. Sham: 0.17 [−0.39–0.72]	**NA**	**BOLD Signal** NA	No significant difference in alcohol craving was observed following 1 active cTBS session compared to sham. cTBS significantly ↓ evoked BOLD signal in left OFC, insula, and lateral sensorimotor cortex.
Jansen et al. [60]	*N* = 39	A randomized, single-blind, sham-controlled study with recently detoxified alcohol-dependent participants	1 Active *OR* 1 Sham Session **Right DLPFC** *(w/ MRI-neuronavigation)*	110%	10 Hz	Figure-8 Coil	**Craving** Active vs. Sham: −0.31 [−0.78–0.15]	**NA**	**Emotion Regulation** NA	No significant post-treatment effect of active or sham rTMS was observed on alcohol craving, compared to baseline. One active rTMS session reduced self-reported experienced emotions in response to positive and negative images.
**Alcohol**: Multiple Active Stimulation Sessions Total *N* = 458; 11 Studies										
Mishra et al. [61]	*N* = 45	A prospective, single-blind, sham-controlled study with alcohol-dependent participants	10 Active *OR* 10 Sham Sessions **Right DLPFC**	110%	10 Hz	Figure-8 Coil	**Craving** Active vs. Sham: −2.64 [−3.46 – −1.81]	**NA**	**NA**	Active rTMS significantly ↓ alcohol craving compared to sham.
Hoppner et al. [62]	*N* = 19	A randomized, sham-controlled study with alcohol-dependent female participants (14 days after detoxification)	10 Active *OR* 10 Sham Sessions **Left DLPFC**	90%	20 Hz	Figure-8 Coil	**Craving** Active vs. Sham: 1.12 [0.15–2.09]	**NA**	**Depressive Symptoms** Active vs. Sham: −0.15 [−1.06–0.75]	No significant difference in alcohol craving or mood was observed following active rTMS, compared to sham and baseline.
Ceccanti et al. [63]	*N* = 18	A randomized, double-blind, sham-controlled study with alcohol-dependent male participants	10 Active *OR* 10 Sham Sessions of deep TMS (dTMS) **mPFC**	120%	20 Hz	H-coil (H-1)	**Craving** Active vs. Sham: −1.64 [−2.71 – −0.57]	**Consumption** Active vs. Sham: −2.07 [−3.21– −0.92]	**Dopamine Pathway Modulation** NA	Active dTMS significantly ↓ alcohol craving compared to sham. Significant ↓ in cortisolemia and prolactinemia was observed following active dTMS compared to sham, suggesting dopamine increase.
Girardi et al. [64]	*N* = 20	An open-label, double-blind, sham-controlled study with alcohol-dependent participants with dysthymia receiving concurrent standard detoxification treatment	20 Active Sessions of dTMS with SDT *OR* SDT alone **Bilateral DLPFC**	120%	20 Hz	H-coil (H-1)	**Craving** Active vs. Sham: −1.49 [−2.48 – −0.50]	**NA**	**Depressive Symptoms** Active vs. Sham: −1.26 [−2.22 – −0.30]	Combined pharmacotherapy and dTMS resulted in a significant ↓ in alcohol craving and depressive symptoms
Addolorato et al. [65]	*N* = 11	A randomized, sham-controlled study with alcohol-dependent participants	12 Active *OR* 12 Sham Sessions **Bilateral DLPFC**	100%	10 Hz	Figure-8 Coil	**NA**	**Consumption** Active vs. Sham: 0.16 [−1.03–1.35]	**DAT Availability** NA	Active rTMS significantly ↓ striatal DAT availability and alcohol consumption compared to baseline.
Perini et al. [66]	*N* = 56	A randomized, double-blind, sham-controlled study with alcohol-dependent participants	15 Active *OR* 15 Sham Sessions of dTMS **Insula**	120%	10 Hz	H-coil (H-8)	**Craving** Active vs. Sham: 1.28 [0.71–1.86]	**Consumption** Active vs. Sham: −0.52 [−1.05–0.01]	**NA**	No significant difference in alcohol craving or consumption was observed following active dTMS compared to sham stimulation.
Raikwar et al. [67]	*N* = 60	A randomized, single-blind, sham-controlled study with alcohol-dependent male participants	10 Active *OR* 10 Sham Sessions **Left DLPFC**	120%	10 Hz	Figure-8 Coil	**Craving** Active vs. Sham: −0.19 [−0.70–0.31]	**NA**	**NA**	No significant difference in alcohol craving was observed following active rTMS compared to sham stimulation.
Harel et al. [68]	*N* = 51	A randomized, double-blind, sham-controlled study with recently abstinent, alcohol-dependent participants.	15 Active + 5 Maintenance *OR* 15 Sham + 5 Maintenance Sessions of dTMS **mPFC and ACC**	100%	10 Hz	H-coil (H-7)	**Craving** Active vs. Sham: −2.39 [−3.15 – −1.63]	**Consumption** Active vs. Sham: −2.61 [−3.39 – −1.82]	**NA**	Active dTMS significantly ↓ pHDD and alcohol craving compared to sham.
Zhang et al. [69]	*N* = 48	A randomized, double-blind, sham-controlled study with alcohol-dependent participants.	10 Active *OR* 10 Sham Sessions **Left DLPFC**	110%	20 Hz	Figure-8 Coil	**Craving** Active vs. Sham: −2.22 [−2.99 – −1.45]	**Consumption** Active vs. Sham: −1.84 [−2.57 – −1.12]	**NA**	Active rTMS significantly ↓ days of heavy drinking and alcohol craving compared to sham.
Hoven et al. [70]	*N* = 80	A randomized, dingle-blind, sham-controlled study with recently abstinent, alcohol-dependent participants.	10 Active *OR* 10 Sham Sessions **Right DLPFC**	110%	10 Hz	Figure-8 Coil	**Craving** Active vs. Sham: −0.16 [−0.59–0.28]	**NA**	**Abstinent Days Over 6-months** Active vs. Sham: −0.13 [−0.56–0.30]	No significant difference in number of abstinent days over 6-months or alcohol craving was observed following active rTMS compared to sham stimulation.
McCalley et al. [71]	*N* = 50	A randomized, double-blind, sham-controlled study with alcohol-dependent participants.	10 Active *OR* 10 Sham Sessions of **cTBS** **mPFC**	110%	3-pulse 50 Hz bursts given every 200 ms (at 5 Hz)	Figure-8 Coil	**Craving** Active vs. Sham: −4.36 [−5.38 – −3.34]	**NA**	**Alcohol Cue Reactivity** NA **Sobriety** NA	Active cTBS significantly ↓ brain reactivity to alcohol cues and alcohol craving compared to sham. No significant difference in sobriety was observed, though active cTBS participants were three times as likely to remain sober at the 3-month follow-up.
**Tobacco**: Single Active Stimulation Session Total *N* = 52; 4 Studies										
Rose et al. [83]	*N* = 15	A repeated measure, counterbalanced design with tobacco-dependent participants ( > 15 CPD)	1 Active (1 Hz) *AND* 1 Active (10 Hz) *AND* 1 Control (MOC) Session **SFG**	90%	LF: 1 Hz *and* HF: 10 Hz	Figure-8 Coil	**Cue-induced Craving** **(Neutral Cue)** 10 Hz vs. Sham: −6.13 [−7.84 – −4.42] **Cue-induced Craving** **(Smoking Cue)** 10 Hz vs. Sham: −2.37 [−3.31 – −1.44]	**NA**	**NA**	10-Hz SFG rTMS significantly ↓ cigarette craving following neutral cue presentation, relative to 1-Hz SFG and MOC. Conversely, 10-Hz rTMS significantly ↑ cigarette craving after presentation of smoking cues.
Li et al. [74]	*N* = 14	A randomized, double-blind, sham-controlled crossover study with tobacco-dependent participants	1 Active *AND* 1 Sham Session **Left DLPFC**	100%	10 Hz	Figure-8 Coil	**Subjective Craving** Active vs. Sham: −1.02 [−1.81 – −0.24] **Cue-induced Craving** Active vs. Sham: −0.27 [−1.01–0.48]	**NA**	**NA**	Active rTMS, but not sham, significantly ↓ subjective craving and cue-induced tobacco craving compared to baseline.
Pripfl et al. [75]	*N* = 11	A sham-controlled, crossover study with tobacco-dependent participants	1 Active *AND* 1 Sham Session **Left DLPFC** *(w/ MRI-neuronavigation)*	90%	10 Hz	Figure-8-Coil	**Cue-induced Craving** Active vs. Sham: −0.26 [−1.10–0.58]	**NA**	**EEG Delta Power** Active vs. Sham: −0.23 [−1.06–0.61]	Active rTMS significantly ↓ EEG delta power and tobacco craving compared to sham.
Li et al. [72]	*N* = 10	A sham-controlled, counterbalanced, crossover study with tobacco-dependent participants	1 Active *AND* 1 Sham Session **Left DLPFC**	100%	10 Hz	Figure-8 Coil	**Craving** Active vs. Sham: −0.04 [−0.92–0.83]	**NA**	**mOFC and NAc Activity** NA	Active rTMS significantly ↓ activity in mOFC and NAc. No significant difference in tobacco craving was observed.
**Tobacco**: Multiple Active Stimulation Sessions Total *N* = 729; 12 Studies										
Eichhammer et al. [76]	*N* = 14	A double-blind, sham controlled, crossover study with tobacco-dependent participants	2 Active *AND* 2 Sham Sessions (on a single day) **Left DLPFC**	90%	20 Hz	Figure-8 Coil	**Craving** NA	**Consumption** Active vs. Sham: −0.38 [−1.13–0.37]	**NA**	Active rTMS significantly ↓ cigarette consumption compared to sham. No significant difference in tobacco craving was observed.
Amiaz et al. [77]	*N* = 48	A randomized, double-blind, sham-controlled study with tobacco-dependent participants	10 Active + 6 Maintenance *OR* 10 Sham + 6 Maintenance Sessions **Left DLPFC** *(with neutral or smoking cue-provocation)*	100%	10 Hz	Figure-8 Coil	**Craving** Active (Neutral Cue Provocation) vs. Sham: −0.12 [−0.86–0.63] Active (Smoking Cue Provocation) vs. Sham: −3.26 [−4.48 – −2.03]	**Consumption** Active (Neutral Cue Provocation) vs. Sham: −1.01 [−1.80 – −0.23] Active (Smoking Cue Provocation) vs. Sham: −2.99 [−4.16 – −1.82]	**Dependence** Active (Neutral Cue Provocation) vs. Sham: −1.93 [−2.83 – −1.03] Active (Smoking Cue Provocation) vs. Sham: −2.45 [−3.51 – −1.39]	Active rTMS significantly ↓ cigarette consumption and nicotine dependence compared to sham. However, this was not maintained 6-months post-treatment. Craving was decreased only in the group that received active rTMS following smoking cue provocation.
Wing et al. [78]	*N* = 15	A randomized, double-blind, sham-controlled study with tobacco-dependent participants with comorbid schizophrenia (SCZ)	20 Active *OR* 20 Sham Sessions **Bilateral DLPFC**	90%	20 Hz	Figure-8 Coil	**Craving** Active vs. Sham: −0.43 [−1.47–0.62]	**NA**	**NA**	Active rTMS significantly ↓ tobacco craving compared to sham and baseline.
Dieler et al. [79]	*N* = 74	A randomized, sham controlled study with tobacco-dependent participants	4 Active *OR* 4 Sham Sessions of **iTBS** with concurrent CBT **Right DLPFC**	80%	50 Hz	Figure-8 Coil	**Craving** Active vs. Sham: 0.32 [−0.13–0.78]	**NA**	**Abstinence** NA	iTBS with adjunct CBT produced ↑ abstinence rates at 3 months compared to sham. No significant effect on craving was observed
Dinur-Klein et al. [84]	*N* = 115	A prospective, randomized, sham-controlled study with tobacco-dependent participants ( > 20 CPD)	13 Active *OR* 13 Sham Sessions of dTMS **Lateral PFC and Insula** *(with or without smoking cue-provocation)*	120%	LF: 1 Hz *or* HF: 10 Hz	H-coil (H-ADD)	**NA**	**Consumption** 10 Hz vs. Sham: −5.25 [−6.29 – −4.21]	**Abstinence** NA	10-Hz dTMS significantly ↓ cigarette consumption and nicotine dependence compared to low frequency dTMS and sham. The combination of dTMS with smoking cue provocation enhanced this reduction in consumption. No significant difference in abstinence rates was observed between groups.
Prikryl et al. [80]	*N* = 35	A randomized, double-blind, sham-controlled study with tobacco-dependent male participants with comorbid SCZ	15 Active *OR* 15 Sham Sessions **Left DLPFC**	110%	10 Hz	Figure-8 Coil	**NA**	**Consumption** Active vs. Sham: −0.44 [−1.11–0.24]	**NA**	Active rTMS significantly ↓ cigarette consumption compared to sham.
Trojak et al. [81]	*N* = 37	A prospective, randomized, sham-controlled study with tobacco-dependent participants receiving concurrent nicotine replacement therapy (NRT)	10 Active *OR* 10 Sham Sessions with concurrent NRT **Right DLPFC** *(w/ MRI-neuronavigation)*	120%	1 Hz	Figure-8 Coil	**Craving** Active vs. Sham: 0.11 [−0.53–0.76]	**NA**	**Abstinence** NA	Active rTMS combined with NRT produced significantly ↑ abstinent participants. However, this was not maintained at follow-up (12 weeks). No lasting effects on tobacco craving was observed.
Kozak et al. [73]	*N* = 27	A double-blind, sham-controlled, crossover study with tobacco-dependent participants with & without comorbid SCZ	6 Active *AND* 6 Sham Sessions **Bilateral DLPFC**	90%	20 Hz	Figure-8 Coil	**Craving** Active vs. Sham: 0.18 [−0.59–0.95]	**NA**	**Cognition** NA	Active rTMS had no significant effects on tobacco craving, tobacco withdrawal, or cognitive outcomes, when compared to sham.
Abdelrahman et al. [82]	*N* = 40	A randomized, double-blind, sham-controlled study with tobacco-dependent participants	10 Active *OR* 10 Sham Sessions **Left DLPFC**	80%	20 Hz	Figure-8 Coil	**Craving** Active vs. Sham: −0.96 [−1.62 – −0.31]	**Consumption** Active vs. Sham: −1.62 [−2.33 – −0.91]	**Depressive Symptoms** Active vs. Sham: −1.26 [−1.93 – −0.58]	Active rTMS significantly ↓ cigarette consumption and tobacco craving compared to sham. Significant improvement in depressive symptoms was observed following active rTMS treatment compared to sham and baseline.
Zangen et al. [37]	*N* = 262	A multicenter, randomized, double-blind, sham-controlled study with tobacco-dependent participants	15 Active + 3 Maintenance *OR* 15 Sham +3 Maintenance Sessions of dTMS **Bilateral PFC and Insula**	120%	10 Hz	H-coil (H-4)	**Craving** Active vs. Sham: −3.42 [−3.80 – −3.04]	**NA**	**Continuous Quit Rate (CQR)** Active vs. Sham: 2.12 [1.82–2.42]	Active dTMS produced a ↑ CQR compared to sham. Active dTMS significantly ↓ tobacco craving compared to sham.
Ibrahim et al. [86]	*N* = 42	A randomized, double-blind, sham-controlled study with tobacco-dependent participants receiving concurrent varenicline treatment	20 Active *OR* 20 Sham Sessions of dTMS **Insular Cortex**	120%	10 Hz	H-coil (H-11)	**Craving** Active vs. Sham: −0.35 [−1.03–0.33]	**Consumption** Active vs. Sham: −0.12 [−0.79–0.56]	**Abstinence** NA	Active dTMS had no significant effect on craving or consumption at the end of stimulation treatment (Week 4). However, at the end of varenicline treatment (Week 12), smokers in the active group had significantly higher abstinence rates than those who received sham (82.4% vs. 30.7%).
Moeller et al. [85]	*N* = 20	A randomized, double-blind, sham-controlled study with tobacco-dependent participants with comorbid SCZ	15 Active *OR* 15 Sham Sessions of dTMS **Bilateral PFC and Insula**	120%	10 Hz	H-coil (H-4)	**NA**	**Self-administration** NA	**Psychiatric Symptoms** NA	Active dTMS significantly ↑ the latency for patients to smoke their first cigarette, compared to sham. Active dTMS produced a stepwise reduction in psychotic symptoms overtime.
**Cannabis**: Single Active Stimulation Session Total *N* = 14; 1 Study										
Sahlem et al. [87]	*N* = 14	A randomized, double-blind, sham-controlled, crossover study with cannabis-dependent participants	1 Active *AND* 1 Sham Session **Left DLPFC**	110%	10 Hz	Figure-8 Coil	**Craving** Active vs. Sham: 0.42 [−0.33–1.17]	**NA**	**Retention** NA	rTMS can be safely administered to cannabis-dependent patients and is well tolerated. No significant reduction in craving was observed following active rTMS compared to sham.
**Cannabis**: Multiple Active Stimulation Sessions Total *N* = 19; 1 Study										
Bidzinski et al. [88]	*N* = 19	A randomized, double-blind, sham-controlled parallel groups study with cannabis-dependent participants with comorbid SCZ	20 Active *OR* 20 Sham Sessions **Bilateral DLPFC**	90%	20 Hz	Figure 8 Coil	**NA**	**Consumption** Active vs. Sham: −0.34 [−1.30–0.62]	**Psychiatric Symptoms** NA	Non-significant reduction in cannabis consumption and an improvement in positive symptoms of psychosis were observed following active rTMS compared to sham. A trend towards a greater reduction in craving was observed following active rTMS.
**Cocaine**: Single Active Stimulation Sessions Total *N* = 25; 1 Study										
Hanlon et al. [59]	*N* = 24	A single-blind, sham-controlled, crossover study with cocaine-dependent participants	1 Active *AND* 1 Sham Session of **cTBS** **Left Frontal Pole**	110%	3-pulse bursts presented at 5 Hz	Figure-8 Coil	**Craving** Active vs. Sham: 0.01 [−0.55–0.58]	**NA**	**BOLD Signal** NA	No significant difference in cocaine craving was observed following 1 active cTBS session compared to sham. cTBS significantly ↓ evoked BOLD signal in the caudate, accumbens, anterior cingulate, orbitofrontal (OFC) and parietal cortex.
**Cocaine**: Multiple Active Stimulation Sessions Total *N* = 202; 5 Studies										
Bolloni et al. [89]	*N* = 10	A randomized, double-blind, sham-controlled study with cocaine-dependent participants	12 Active *OR* 12 Sham Sessions of dTMS **Bilateral PFC**	100%	10 Hz	H-coil (H-1)	**NA**	**Consumption** Active vs. Sham: −1.46 [−2.50 – −0.41]	**NA**	Active dTMS did not significantly affect cocaine consumption compared to sham. However, a decreasing trend in consumption between baseline and 6-months post-dTMS was observed in the active group.
Terraneo et al. [90]	*N* = 32	A randomized, open-label study with cocaine-dependent participants.	8 Active Sessions *OR* SDT only **Left DLPFC** *(w/ MRI-neuronavigation)*	100%	15 Hz	Figure-8 Coil	**Craving** Active vs. Sham: −2.24 [−3.17 – −1.30]	**NA**	**Relapse** NA	Active rTMS significantly ↑ clean urine screens and ↓ cocaine craving compared to sham.
Martinez et al. [91]	*N* = 18	A randomized, sham-controlled study with cocaine-dependent participants.	13 Active *OR* 13 Sham Sessions of dTMS **mPFC and dACC**	90% − 110%	LF:1 Hz *or* HF:10 Hz	H-coil (H-7)	**Craving** NA	**Self-administration** NA	**NA**	10-Hz dTMS significantly ↓ cocaine self-administration, relative to 1-Hz dTMS and sham. No significant effect on craving was observed.
Lolli et al. [92]	*N* = 62	A randomized, blinded, sham-controlled study with cocaine-dependent participants.	15 Active *OR* 15 Sham Sessions **Left DLPFC**	100%	15 Hz	Figure-8 Coil	**Cue-induced Craving** Active vs. Sham: 0.24 [−0.43–0.92]	**NA**	**Time to Urine Negativation** NA	No significant difference between the active and sham rTMS groups in the time to urine negativization. However, cue-induced cocaine craving significantly ↓ in the active rTMS group only.
Martinotti et al. [93]	*N* = 80	A randomized, blinded, sham-controlled, multicentre study with cocaine-dependent participants.	20 Active + 24 Maintenance *OR* 20 Sham + 24 Maintenance Sessions of accelerated (twice daily) rTMS **Left DLPFC**	100%	15 Hz	Figure-8 Coil	**Craving** Active vs. Sham: −0.21 [−0.68–0.27]	**NA**	**% of Negative Urine Tests** NA	There were no significant differences in cocaine craving and consumption between active and sham rTMS groups.
**Methamphetamine**: Single Active Stimulation Session Total *N* = 18; 1 Study										
Li et al. [94]	*N* = 18	A single-blind, sham-controlled, crossover study with methamphetamine-dependent participants and matched healthy controls.	1 Active *AND* 1 Sham Session **Left DLPFC**	100%	1 Hz	Figure-8 Coil	**Cue-induced Craving** Active vs. Sham: 4.42 [2.79–6.04]	**NA**	**NA**	Active rTMS significantly ↑ cue-induced craving for methamphetamine in MUD participants compared to sham. This effect was not observed in healthy controls.
**Methamphetamine**: Multiple Active Stimulation Sessions Total *N* = 501; 7 Studies										
Su et al. [95]	*N* = 30	A randomized, double-blind, sham-controlled study with methamphetamine- dependent participants.	5 Active *OR* 5 Sham Sessions **Left DLPFC**	100%	10 Hz	Figure-8 Coil	**Cue-induced Craving** Active vs. Sham: −0.65 [−1.39–0.08]	**NA**	**Cognition** NA	Active rTMS significantly ↓ cue-induced craving for methamphetamine compared to sham. Significant improvement in cognition post-rTMS.
Liang et al. [96]	*N* = 48	A double-blind, randomized, sham-controlled study with methamphetamine- dependent male participants.	10 Active *OR* 10 Sham Sessions **Left DLPFC**	100%	10 Hz	Not Mentioned	**Cue-induced Craving** Active vs. Sham: −6.84 [−8.36 – −5.33]	**NA**	**Depressive Symptoms** NA	Active rTMS significantly ↓ cue-induced craving for methamphetamine, as well as improved depressive symptoms and sleep quality, compared to sham.
Liu et al. [97]	*N* = 90	A randomized, between-subjects study with methamphetamine-dependent female participants.	20 Active Sessions *OR* No Treatment **Left DLPFC**	100%	10 Hz	Figure-8 Coil	**Craving** Active vs. No Treatment: −5.34 [−6.23 – −4.46]	**NA**	**NA**	rTMS treatment significantly ↓ methamphetamine craving, with the effect lasting 30 days post-treatment.
Chen et al. [98]	*N* = 74	A between groups, randomized, sham-controlled study with methamphetamine- dependent participants.	10 Active **Left DLPFC iTBS (A)** *OR* 10 Active **Left vmPFC cTBS (B)** *OR* 10 Active **Left DLPFC iTBS** ***and*** **Left vmPFC cTBS (C)** *OR* 10 Sham Sessions	100% (iTBS) *or* 110% (cTBS)	3-pulse 50 Hz bursts given every 200 ms (at 5 Hz)	Figure-8 Coil	**Cue-induced Craving** A vs. Sham: −0.88 [−1.55 – −0.20] B vs. Sham: −1.31 [−2.02 – −0.60] C vs. Sham: −1.53 [−2.25 – −0.81]	**NA**	**Depressive Symptoms** NA	Cue-induced craving for methamphetamine was significantly ↓ in all three active TBS groups compared to sham. Combined iTBS and cTBS treatment significantly improved depressive and withdrawal symptoms compared to sham and baseline.
Su et al. [99]	*N* = 126	A randomized, double-blind, sham-controlled study with methamphetamine- dependent participants.	20 Active *OR* 20 Sham Sessions of **iTBS** **Left DLPFC**	100%	3-pulse 50 Hz bursts given every 200 ms (at 5 Hz)	Figure-8 Coil	**Craving** Active vs. Sham: −1.14 [−1.52 – −0.76]	**NA**	**Cognition** NA	Active iTBS significantly ↓ methamphetamine craving and improved cognition and sleep quality, compared to sham.
Su et al. [100]	*N* = 60	A randomized, double-blind, sham-controlled study with methamphetamine- dependent patients	20 Active *OR* 20 Sham Sessions of **iTBS** **Left DLPFC**	100%	3-pulse 50 Hz bursts given every 200 ms (at 5 Hz)	Figure-8 Coil	**Cue-induced Craving** Active vs. Sham: −1.01 [−1.55 – −0.47]	**NA**	**Functional Connectivity** NA	Active iTBS significantly ↓ methamphetamine craving compared to sham. A significant ↑ in functional connectivity between left DLPFC and inferior parietal lobule was observed following iTBS, which correlated with craving reduction.
Yuan et al. [101]	*N* = 73	A randomized, double-blind, sham-controlled study with methamphetamine- dependent male participants	10 Active *OR* 10 Sham Sessions **Left PFC**	100%	1 Hz	Figure-8 Coil	**Cue-induced Craving** Active vs. Sham: −0.21 [−0.67–0.25]	**NA**	**Impulse Inhibition** NA	Significant ↓ in cue-induced craving following active rTMS compared to sham and baseline. Significant improvement in accuracy and reaction time following single session of rTMS, maintained after 10 sessions and at 3 weeks post-treatment.
**Opioid**: Multiple Active Stimulation Sessions Total *N* = 239; 4 Studies										
Shen et al. [103]	*N* = 20	A randomized, sham-controlled, study with heroin-dependent male participants	5 Active *OR* 5 Sham Sessions **Left DLPFC**	100%	10 Hz	Figure-8 Coil	**Cue-induced Craving** Active vs. Sham: −3.12 [−4.43 – −1.82]	**NA**	**NA**	Active rTMS caused a significant ↓ in craving scores after presentation of heroin-related cues, compared to sham.
Liu et al. [104]	*N* = 99	A randomized, double-blind, sham-controlled study with heroin-dependent male participants	20 Active (1 Hz) *OR* 20 Active (10 Hz) Sessions *OR* No Treatment **Left DLPFC**	100%	LF: 1 Hz *or* HF: 10 Hz	Figure-8 Coil	**Cue-induced Craving** 1 Hz vs. No Treatment: −0.57 [−1.04 – −0.09] 10 Hz vs. No Treatment: −0.71 [−1.18 – −0.25]	**NA**	**NA**	Both of the active rTMS groups had a significant ↓ in cue-induced heroin craving compared to no treatment. The effects were consistent 60 days following treatment cessation.
Li et al. [105]	*N* = 100	A retrospective, sham-controlled study with morphine-dependent participants receiving concurrent occupational therapy (OT)	40 Active *OR* 40 Sham Sessions with concurrent OT **Left DLPFC**	100%	20 Hz	Figure-8 Coil	**Craving** Active vs. Sham: −1.79 [−2.25 – −1.32]	**NA**	**Depressive Symptoms** NA	Active rTMS significantly ↓ in morphine craving and improved depressive symptoms, compared to baseline and sham.
Tsai et al. [102]	*N* = 20	A randomized, double-blind, sham-controlled study with heroin-dependent male participants, receiving concurrent methadone maintenance therapy (MMT)	11 Active *OR* 11 Sham Sessions with concurrent MMT **Left DLPFC**	100%	15 Hz	Figure-8 Coil	**Craving** Active vs. Sham: 1.23 [0.27–2.19]	**Consumption** NA	**Depressive Symptoms** NA	Active rTMS had no significant effect on heroin craving or heroin consumption, compared to sham. However, a significant improvement in depressive symptoms was observed post-treatment.

Bold values have been used to highlight the outcome of interest and the brain region targeted, to improve clarity. Substance use disorder investigated is also shown in bold.

**Table 2:** Transcranial Direct Current Stimulation (tDCS) [Total *N* = 1582; Total Studies = 36].

Author	Sample Size	Study Design	# of Sessions & Targeted Region	Active Stimulation Intensity & Duration	Craving Effect Size (Hedge’s *g*) [95% CI]	Consumption Effect Size (Hedge’s *g*) [95% CI]	Other Outcome(s) Effect Size (Hedge’s *g*) [95% CI]	Results
**Alcohol**: Single Active Stimulation Session Total *N* = 178; 5 Studies								
Boggio et al. [106]	*N* = 13	A randomized, sham-controlled, crossover study with alcohol-dependent participants.	1 Session of ***An+ Right***, Ca- Left **DLPFC** *AND* 1 Session of ***An+ Left***, Ca- Right **DLPFC** *AND* 1 Session of Sham	2 mA for 20 min	**Craving** An+ Right vs. Sham: −1.96 [−2.83 – −0.98] An+ Left vs. Sham: −1.05 [−1.83 – −0.20]	**NA**	**NA**	Both An+ right and An+ left tDCS significantly ↓ alcohol craving, compared to sham and baseline. These results were maintained when presented with alcohol cues.
Nakamura-Palacios et al. [115]	*N* = 49	A randomized, sham-controlled, crossover study with alcohol-dependent participants.	1 Session of ***An+ Left*** **DLPFC**, Ca- **CSDA** *OR* 1 Sham Session	1 mA for 10 min	**Craving** Active vs. Sham: −0.16 [−1.29–0.98]	**NA**	**P3 Amplitude** NA	ERPs indicated an ↑ in P3 amplitude to alcohol related sounds in the active tDCS group compared to sham. No significant effect of treatment on alcohol craving was observed.
den Uyl et al. [109]	*N* = 41	A randomized, sham-controlled study with alcohol-dependent participants.	1 Session of ***An+ Left*** **DLPFC**, Ca- **CSOA** *OR* l Session of An+ **IFG** (F7xCz), Ca^**--**^ **IFG** (FzxT3) *OR* 1 Sham Session	1 mA for 10 min	**Craving** DLPFC vs. Sham: −0.15 [−0.92–0.62] IFG vs. Sham: 0.19 [−0.57–0.95]	**NA**	**Response Bias** NA	Active tDCS over the left DLPFC significantly ↓ alcohol craving, compared to sham and IFG stimulation. No significant effect of tDCS on response bias was observed.
Wietschorke et al. [117]	*N* = 30	A randomized, double-blind, sham-controlled study with alcohol-dependent participants.	1 Session of ***An+ Right***, Ca- Left **DLPFC** *OR* 1 Sham Session	1 mA for 20 min	**Craving** Active vs. Sham: −0.55 [−1.26–0.20]	**NA**	**Alcohol Cue Reactivity** NA	Active tDCS significantly ↓ alcohol cue reactivity and alcohol craving, compared to sham.
Vanderhasselt et al. [116]	*N* = 45	A randomized, double-blind, sham-controlled study with alcohol-dependent participants.	1 Session of ***An+ Right***, Ca- Left **DLPFC** *AND* 1 Sham Session	2 mA for 20 min	**NA**	**Consumption** NA	**Reward-triggered Approach Bias** NA	Active tDCS significantly ↓ reward-triggered approach bias and alcohol consumption, compared to sham.
**Alcohol**: Multiple Active Stimulation Sessions Total *N* = 556; 9 Studies								
da Silva et al. [108]	*N* = 13	A randomized, sham-controlled study with alcohol-dependent male participants.	5 Sessions of ***An+ Left***, Ca- Right **DLPFC** *OR* 5 Sessions of Sham	2 mA for 20 min	**Craving** Active vs. Sham: −1.87 [−3.17 – −0.56]	**NA**	**Relapse** NA	A significant ↑ in relapse rates was observed following active tDCS (66.7%) compared to sham (14.3%). However, active tDCS significantly ↓ alcohol craving.
Klauss et al. [113]	*N* = 33	A randomized, sham-controlled study with alcohol dependent participants.	5 Sessions ***An+ Right***, Ca- Left **DLPFC** *OR* 5 Sessions of Sham	2 mA for 13 min	**Craving** Active vs. Sham: −0.16 [−0.84–0.53]	**NA**	**Relapse** NA	A significant ↓ in relapse rates was observed 6-months following active tDCS (50%) compared to sham (88.2%). No significant effect on alcohol craving was observed.
den Uyl et al. [110]	*N* = 78	A randomized, double-blind, sham-controlled, 2-by-2 factorial design study with alcohol dependent participants receiving concurrent cognitive bias modification (CBM).	3 Sessions ***An+ Left*** **DLPFC**, Ca- **CSOA** with ***active CBM*** **(A)** *OR* 3 Sessions ***An+ Left*** **DLPFC**, Ca- **CSOA** with control CBM **(B)** *OR* 3 Sessions of Sham with ***active CBM*** **(C)** *OR* 3 Sessions of Sham with control CBM **(D)**	1 mA for 15 min	**Cue-induced Craving** Active vs. Sham: 0 [−0.44–0.44]	**NA**	**Approach Bias** NA	A significant ↓ in cue-induced alcohol craving, but not overall craving, was observed in the active tDCS groups compared to sham. There were no enhancement effects of tDCS on CBM.
den Uyl et al. [111]	*N* = 91	A randomized, double-blind, sham-controlled study with alcohol-dependent participants receiving concurrent CBM.	4 Sessions ***An+ Left*** **DLPFC**, Ca- **CSOA** with ***active CBM*** *OR* 4 Sessions ***An+ Left*** **DLPFC**, Ca- **CSOA** without CBM *OR* 4 Sessions of Sham with ***active CBM***	2 mA for 20 min	**Craving** Active/CBM+ vs. Sham: 1.18 [0.63–1.73] Active/CBM- vs. Sham: −0.30 [−0.80–0.21]	**NA**	**Abstinence** Active/CBM+ vs. Sham: 0.26 [−0.25–0.77] Active/CBM- vs. Sham: 0.24 [−0.27–0.74]	Active tDCS had no significant effect on abstinence duration at 3- or 6-months post-treatment. Alcohol craving ↓ overtime in all conditions. There were no enhancement effects of tDCS on CBM.
den Uyl et al. [112]	*N* = 83	A randomized, double-blind, sham-controlled, 2-by-2 factorial design study with alcohol dependent participants receiving concurrent attentional bias modification (ABM).	4 Sessions ***An+ Left***, Ca- Right **DLPFC** with ***active ABM*** **(A)** *OR* 4 Sessions ***An+ Left***, Ca- Right **DLPFC** with control ABM **(B)** *OR* 4 Sessions of Sham with ***active ABM*** **(C)** *OR* 4 Sessions of Sham with control ABM **(D)**	2 mA for 20 min	**Craving** A vs. C: −0.49 [−1.11–0.13] B vs. D: −0.74 [−1.36 – −0.11]	**NA**	**Attentional Bias** NA	Active tDCS had no significant effect on attentional bias, alcohol craving, or relapse. There was no evidence of a beneficial effect of active tDCS, ABM, or the combination.
Klauss et al. [114]	*N* = 49	A randomized, double-blind, sham-controlled study with alcohol-dependent participants.	10 Sessions of ***An+ Right***, Ca- Left **DLPFC** *OR* 10 Sessions of Sham	2 mA for 20 min	**Craving** Active vs. Sham: −0.58 [−1.17–0.02]	**NA**	**Relapse** NA	A ↓ in alcohol craving was observed following active tDCS and sham. However, the change in craving was significant only in the active tDCS group. Active tDCS significantly ↓ relapse rates at 3-months post-treatment.
Claus et al. [107]	*N* = 79	A randomized, double-blind, sham-controlled, 2-by-2 factorial design study with alcohol dependent participants receiving concurrent CBM.	4 Sessions An+ Right **IFG**, Ca- **Contralateral Upper Arm** with ***active CBM*** (**A**) *OR* 4 Sessions An+ Right **IFG**, Ca- **Contralateral Upper Arm** with control CBM (**B**) *OR* 4 Sessions of Sham with ***active CBM*** (**C**) *OR* 4 Sessions of Sham with control CBM (**D**)	2 mA for 20 min	NA	**Consumption** A vs. C: −0.25 [−0.89–0.39] B vs. D: 0.22 [−0.40–0.84]	**Approach Bias** NA	There was no significant effect of active tDCS, CBM, or CBM-tDCS interaction on alcohol approach bias. While active tDCS displayed a trend towards a reduction in alcohol consumption, the difference was not significant.
Witkiewitz et al. [118]	*N* = 84	A randomized, double-blind, sham-controlled study with alcohol-dependent participants receiving concurrent mindfulness-based relapse prevention (MBRP).	8 Sessions of An+ Right **IFG**, Ca- Left **Upper Arm** with active MBRP *OR* 8 Sessions of Sham with active MBRP	2 mA for 30 min	**Craving** Active vs. Sham: 0.07 [−0.36–0.50]	**Consumption** Active vs. Sham: −0.14 [−0.57–0.29]	**NA**	There was no significant difference in post-treatment alcohol consumption and craving between active and sham tDCS.
Dubuson et al. [119]	*N* = 125	A randomized, double-blind, sham-controlled, 2-by-2 factorial design study with alcohol dependent participants receiving concurrent inhibitory control training (ICT).	5 Session of ***An+ Right***, Ca- Left **DLPFC** with ***active ICT*** **(A)** *OR* 5 Session of ***An+ Right***, Ca- Left **DLPFC** with control ICT **(B)** *OR* 5 Sessions of Sham with ***active ICT*** **(C)** *OR* 5 Sessions of Sham with control ICT **(D)**	2 mA for 20 min	**Craving** A vs. C: 0.48 [−0.09–1.04] B vs. D: 0.12 [−0.43–0.67]	**NA**	**Abstinence** NA	Active tDCS (A, B) significantly ↑ abstinence rates at 2-week follow up compared to sham (C, D), independent of ICT. Active tDCS with concurrent ICT (A) produced the highest abstinence rates. No treatment effects on craving were observed.
**Tobacco**: Single Active Stimulation Session Total *N* = 157; 6 Studies								
Fregni et al. [125]	*N* = 24	A randomized, double-blind, sham-controlled crossover study with tobacco-dependent participants.	1 Session of ***An+ Right***, Ca- Left **DLPFC** *AND* 1 Session of ***An+ Left***, Ca^**-**^ Right **DLPFC** *AND* 1 Session of Sham	2 mA for 20 min	**Craving** An+ Right vs. Sham: −0.47 [−1.04–0.10] An+ Left vs. Sham: −0.38 [−0.95–0.19]	**NA**	**NA**	Active tDCS of both the right and left DLPFC significantly ↓ tobacco craving compared to sham.
Xu et al. [120]	*N* = 24	A single-blind, counterbalanced, sham-controlled study with tobacco-dependent participants.	1 Session of ***An+ Left*** **DLPFC**, Ca- **CSOA** *AND* 1 Session of Sham	2 mA for 20 min	**Craving** Active vs. Sham: 0.05 [−0.52–0.61]	**NA**	**Negative Affect** NA	Compared to sham, active tDCS significantly ↓ negative affect, which is positively correlated with dependence level, but had no effect on tobacco craving.
Meng et al. [121]	*N* = 27	A randomized, counterbalanced, sham-controlled study with tobacco-dependent participants	1 Session of ***An+ Left***, Ca- Right **FPT** *OR* 1 Session of Double An+ Bilateral **Occipital Lobe**, Double Ca- Bilateral **FPT** *OR* 1 Session of Sham	1 mA for 20 min	**NA**	**Consumption** Single Cathodal vs. Sham: −0.16 [−1.08–0.77] Double Cathodal vs. Sham: −2.24 [−3.42 – −1.07]	**Attention Bias** NA	A significant ↓ in cigarette consumption was observed following double cathodal tDCS, compared to sham and single cathodal tDCS. Attention bias showed a declining trend after bilateral cathodal tDCS, but the results were not significantly different from sham.
Kroczek et al. [122]	*N* = 25	A randomized, double-blind, sham-controlled study with tobacco-dependent participants.	1 Session of ***An+ Left*** **DLPFC**, Ca- **OFC** *OR* 1 Session of Sham	2 mA for 15 min	**Cue-induced Craving** Active vs. Sham: 0.54 [−0.26–1.34]	**NA**	**Functional Connectivity** NA	There was no significant difference in cue-induced tobacco craving between active and sham tDCS. Active tDCS significantly ↑ functional connectivity between DLPFC and OFC, compared to sham.
Falcone et al. [123]	*N* = 25	A randomized, double blind, within-subject, counterbalanced, sham-controlled smoking-lapse study with tobacco-dependent participants	1 Session of ***An+ Left*** **DLPFC**, Ca- Right **SOA** *AND* 1 Session of Sham	1 mA for 20 min	**NA**	**Consumption During Session** Active vs. Sham: −0.19 [−0.74–0.37]	**None**	Active tDCS significantly ↑ latency to smoke and ↓ cigarette consumption during the ad libitum smoking session, compared to sham.
Yang et al. [124]	*N* = 32	A single-blind, within-subject, sham-controlled study with tobacco-dependent male participants.	1 Session of ***An+ Right***, Ca- Left **DLPFC** *AND* 1 Session of Sham	1 mA for 30 min	**Cue-induced Craving** Active vs. Sham: −0.22 [−0.71–0.28]	**NA**	**Functional Connectivity** NA	Active tDCS significantly ↓ tobacco craving compared to sham, which correlated with DLPFC-parahippocampal gyrus (PHG) coupling.
**Tobacco**: Multiple Active Stimulation Sessions Total *N* = 291; 5 Studies								
Boggio et al. [126]	*N* = 27	A randomized, sham-controlled study with tobacco-dependent participants.	5 Sessions of ***An+ Right***, Ca- Left **DLPFC** *OR* 5 Sessions of Sham	2 mA for 20 mins	**Subjective Craving** Active vs. Sham: −0.85 [−1.64 – −0.06] **Cue-induced Craving** Active vs. Sham: −1.09 [−1.90 – −0.28]	**Consumption** NA	**NA**	A significant ↓ in subjective tobacco craving, cue-induced tobacco craving, and cigarette consumption was observed following active tDCS compared to sham.
Fecteau et al. [127]	*N* = 12	A randomized, blinded, sham-controlled, crossover study with tobacco-dependent participants.	5 Sessions of ***An+ Right***, Ca- Left **DLPFC** *AND* 5 Sessions of Sham	2 mA for 30 min	**NA**	**Consumption:** Active vs. Sham: −1.83 [−3.18 – −0.48]	**Risk Taking** NA	Active tDCS significantly ↓ cigarette consumption compared to sham, up to four days after the end of the stimulation regiment. No differences in risk taking behavior were observed between treatment conditions.
Smith et al. [128]	*N* = 37	A randomized, double-blind, sham-controlled study with tobacco-dependent participants with comorbid SCZ	5 Sessions of ***An+ Left*** **DLPFC**, Ca- **CSOA** *OR* 5 Sessions of Sham	2 mA for 20 min	**Craving** Active vs. Sham: 0.25 [−0.45–0.93]	**Consumption:** Active vs. Sham: 0.13 [−0.56–0.81]	**Cognition** Active vs. Sham: 0.15 [−0.54–0.83]	Active tDCS significantly ↑ cognitive performance, compared to sham. There was no significant effect of active tDCS on tobacco craving or consumption.
Ghorbani Behnam et al. [129]	*N* = 170	A randomized, sham-controlled study with tobacco-dependent male participants.	Bupropion for 8 weeks **(A)** *OR* 20 Sessions (over 4 weeks) of ***An+ Right***, Ca- Left **DLPFC (B)** *OR* 20 Sessions of Sham (over 4 weeks) **(C)** *OR* 20 Sessions (over 12 weeks) of ***An+ Right***, Ca- Left **DLPFC (D)** *OR* 20 Sessions of Sham (over 12 weeks) **(E)**	2 mA for 20 min	**NA**	**NA**	**Abstinence** NA **Dependence** NA	Longer duration tDCS (D) resulted in the highest abstinence rate at 6 months (25.7%) and was significantly more effective than the shorter duration tDCS (B) and both sham protocols (C, E). Longer duration tDCS (D) significantly ↓ nicotine dependence compared to pharmacotherapy alone (A).
Müller et al. [130]	*N* = 45	A randomized, sham-controlled study with tobacco-dependent participants	5 Sessions of ***An+ Left***, Ca- Right **DLPFC** *OR* 5 Sessions of Sham	2 mA for 20 min	**Craving** Active vs. Sham: −0.90 [−1.52 – −0.27]	**Consumption** Active vs. Sham: −0.15 [−0.74–0.45]	**NA**	There were no significant differences in cigarette craving and consumption between active and sham tDCS groups.
**Cocaine**: Multiple Active Stimulation Sessions Total *N* = 94; 3 Studies								
Batista et al. [131]	*N* = 36	A randomized, double-blind, sham-controlled study with cocaine-dependent male participants.	5 Sessions of ***An+ Right***, Ca- Left **DLPFC** *OR* 5 Sessions of Sham	2 mA for 20 min	**Craving** Active vs. Sham: −0.29 [−0.95–0.37]	**NA**	**NA**	Significant ↓ in craving for crack-cocaine in active tCDS group compared to baseline and sham.
Verveer et al. [132]	*N* = 41	A randomized, sham-controlled study with cocaine-dependent participants.	10 Sessions of ***An+ Right***, Ca^**--**^ Left **DLPFC** *OR* 10 Sessions of Sham	2 mA for 13 min	**Craving** Active vs. Sham: −0.13 [−0.73–0.46]	**NA**	**Relapse** NA	No significant effect of active tDCS on relapse rates or cocaine craving compared to sham. Exploratory analysis indicated a significant ↓ in relapse rates after active tDCS compared to sham in individuals using crack cocaine only.
Gaudreault et al. [133]	*N* = 17	A randomized, double-blind, sham-controlled study with cocaine-dependent participants.	15 Sessions of ***An+ Right***, Ca^**--**^ Left **DLPFC** *OR* 15 Sessions of Sham	2 mA for 20 min	**Craving** Active vs. Sham: −0.14 [−1.20–0.92]	**NA**	**Sleepiness** Active vs. Sham: −1.53	No significant difference in cocaine craving was present between treatment groups, though a decreasing trend in craving was more prominent in the active tDCS group. Active tDCS significantly improved daytime sleepiness compared to sham.
**Methamphetamine**: Single Active Stimulation Session Total *N* = 45; 2 Studies								
Shahbabaie et al. [134]	*N* = 30	A randomized, double-blind, sham-controlled, crossover study with methamphetamine-dependent male participants	1 Session of ***An+ Right*** **DLPFC**, Ca- **CSOA** *AND* 1 Session of Sham	2 mA for 20 min	**Subjective Craving** Active vs. Sham: −0.57 [−1.08 – −0.05] **Cue-induced Craving** Active vs. Sham: 1.12 [0.58–1.67]	**NA**	**NA**	Active tDCS significantly ↓ self-reported craving at rest but ↑ methamphetamine craving during cue-exposure, compared to sham.
Shahbabaie et al. [135]	*N* = 15	A, randomized, double-blind, sham-controlled, crossover study with methamphetamine-dependent male participants	1 Session of ***An+ Right***, Ca- Left **DLPFC** *AND* 1 Session of Sham	2 mA for 20 min	**Craving** Active vs. Sham: −0.85 [−1.60 – −0.26]	**NA**	**Resting State Network Activity** NA	Active tDCS significantly ↓ methamphetamine craving compared to sham. Active tDCS significantly modulated default mode network (DMN), executive control network (ECN), and salience network (SN).
**Methamphetamine**: Multiple Active Stimulation Sessions Total *N* = 150; 3 Studies								
Rohani Anaraki et al. [136]	*N* = 36	A randomized, double-blind, sham-controlled study with methamphetamine- dependent male participants	5 Sessions of ***An+ Right***, Ca- Left **DLPFC** *OR* 5 Sessions of Sham	2 mA for 20 min	**Subjective Craving** Active vs. Sham: −0.12 [−0.83–0.60] **Cue-induced Craving** NA	**NA**	**NA**	Active tDCS significantly ↓ cue-induced methamphetamine craving, but not self-reported instant craving, compared to sham.
Alizadehgoradel et al. [137]	*N* = 39	A randomized, double-blind, sham-controlled study with methamphetamine- dependent male participants	10 Sessions of ***An+ Right***, Ca- Left **DLPFC** *OR* 10 Sessions of Sham	2 mA for 20 min	**Craving** Active vs. Sham: −0.92 [−1.59 – −0.26]	**NA**	**Executive Function** NA	Active tDCS significantly ↓ methamphetamine craving and improved cognitive executive control functions involved in addictive behavior, compared to sham.
Xu et al. [138]	*N* = 75	A randomized, double-blind, sham-controlled study with methamphetamine- dependent female participants	20 Sessions of ***An+ Right***, Ca- Left **DLPFC** with computerized cognitive addiction therapy (CCAT) **(A)** *OR* 20 Sessions of Sham with CCAT **(B)** *OR* No Treatment (**C**)	1.5 mA for 20 min	**Cue-induced Craving** Active vs. Sham: −0.65 [−1.22 – −0.08]	**NA**	**Cognitive Function** NA	Active tDCS with concurrent CCAT significantly ↓ cue-induced methamphetamine craving compared to sham + CCAT and treatment as usual. No significant improvement in attention bias, verbal learning and memory, impulse control, and social cognition was observed.
**Opioid**: Single Active Stimulation Session Total *N* = 20; 1 Study								
Wang et al. [141]	*N* = 20	A randomized, single-blind, sham-controlled study with heroin-dependent male participants	1 Session of Bilateral Ca- **FPT**, An+ **OL** *OR* 1 Session of Sham	1.5 mA for 20 min	**Cue-induced Craving** Active vs. Sham: −2.74 [−3.96 – −1.52]	**NA**	**None**	Active tDCS significantly ↓ cue-induced craving of heroin, compared to sham and baseline.
**Opioid**: Multiple Active Stimulation Sessions Total *N* = 91; 2 Studies								
Taremian et al. [140]	*N* = 60	A randomized, sham-controlled study with opioid-dependent participants receiving concurrent methadone maintenance treatment (MMT)	10 Sessions of ***An+ Right***, Ca- Left **DLPFC** with MMT *OR* 10 Sessions of Sham with MMT *OR* only MMT	2 mA for 20 min	**Craving** Active vs. Sham: −1.13 [−1.80 – −0.46]	**NA**	**Depressive Symptoms** Active vs. Sham: −0.65 [−1.27–0.00]	Active tDCS with concurrent MMT significantly ↓ opium craving, and depressive symptoms compared to sham+MMT and MMT alone.
Eskandari et al. [139]	*N* = 31	A randomized, double-blind, sham-controlled study with opioid-dependent male participants.	10 Sessions of ***An+ Left***, Ca- Right **DLPFC (A)** *OR* 10 Sessions of ***An+ Right***, Ca- Left **DLPFC (B)** *OR* 10 Sessions of Sham	2 mA for 20 min	**Craving** An+ Left vs. Sham: −2.13 [−3.23 – −1.04] An+ Right vs. Sham: −1.39 [−2.34 – −0.43]	**NA**	**Expression Levels of Cytokines** **IL-6** An+ Left vs. Sham: −0.26 [−1.13–0.63] An+ Right vs. Sham: −0.75 [−1.60–0.17] **TNF-ɑ** An+ Left vs. Sham: −0.36 [−1.23–0.54] An+ Right vs. Sham: −0.79 [−1.65–0.13]	Though lower expression levels were present in the active right anodal tDCS group compared to sham, the difference was not statistically significant. Both active tDCS groups and sham significantly ↓ in opium craving, though active tDCS exhibited a greater effect. Active right anodal tDCS significantly ↓ impulsivity compared to sham.

Bold values have been used to highlight the outcome of interest and the brain region targeted, to improve clarity. Substance use disorder investigated is also shown in bold.

**Table 3:** Deep Brain Stimulation (DBS) [Total *N* = 48; Total Studies = 7].

Author	Sample	Study Design	Targeted Region	# of Treatments	Craving, Consumption, and/or Abstinence Effect Size (Hedge’s *g*) [95% CI]	Secondary Outcome(s) Effect Size (Hedge’s *g*) [95% CI]	Results
**Alcohol**: Continuous Active Stimulation Total *N* = 28; 4 Studies							
Voges et al. [142]	*N* = 5	Case reports of alcohol-dependent male participants.	**NAc**	Continuous	**Craving** Post vs. Pre: −3.96 [−6.71 – −1.21] **Abstinence** NA	**None**	A significant ↓ in alcohol craving was observed in all participants. 2/5 patients remained completely abstinent for > 4 years.
Muller et al. [143]	*N* = 5	Case reports of alcohol-dependent male participants.	**NAc**	Continuous	**Craving** Post vs. Pre: −2.11 [−3.66 – −0.57] **Abstinence** NA	**None**	All participants reported a persistent disappearance of alcohol craving. 2/5 participants remained abstinent post-treatment, and the remaining 3 showed a marked reduction of alcohol consumption.
Davidson et al. [144]	*N* = 6	A phase 1 pilot study with alcohol-dependent female participants.	**NAc**	Continuous	**Consumption** Post vs. Pre: −2.01 [−3.40 – −0.62]	**Molecular & Functional Imaging** NA	DBS led to a significant ↓ in alcohol consumption 1-year post-treatment in all participants, as well as a ↓ in alcohol-related compulsivity. Clinical improvements were correlated with a reduction in NAc metabolism and disrupted functional connectivity between the NAc and visual association cortex.
Bach et al. [145]	*N* = 12	A double-blind, randomized, sham-controlled multi-center study with treatment-resistant alcohol-dependent participants.	**NAc**	Continuous	**Craving** Post vs. Pre: −1.36 [−2.62 – −0.11] Active vs. Sham: −0.61 [−1.77–0.55] **Abstinent Days** Post vs. Pre: 1.12 [−0.10–2.33] Active vs. Sham: 0.93 [−0.26–2.12]	**None**	While there was no difference in continuous abstinence between treatment groups at 6-months, active DBS led to a significantly higher proportion of abstinent days over the 6-month period and lower craving scores, compared to sham.
**Tobacco**: Continuous Active Stimulation Total *N* = 10; 1 Study							
Kuhn et al. [146]	*N* = 10	A retrospective, self-report, longitudinal study with tobacco-dependent participants	**NAc**	Continuous	**Dependence** Post vs. Pre: −0.40 [−1.28–0.49]	**None**	3/10 participants quit smoking post-treatment.
**Opioid**: Continuous Active Stimulation Total *N* = 10; 2 Studies							
Kuhn et al. [147]	*N* = 2	Case reports of heroin-dependent participants	**NAc**	Continuous	**Craving** NA	**Depressive Symptoms** NA	A significant ↓ in craving and depressive symptoms was observed 1-year post-DBS in both participants.
Chen et al. [148]	*N* = 8	An open-label study with heroin-dependent participants	**NAc/ALIC**	Continuous	**Craving** Post vs. Pre: −5.75 [−7.97 – −3.53] **Abstinence** NA	**None**	Simultaneous and continuous DBS to the NAc and ALIC led to high abstinence rates (62.5%) and a ↓ in opioid craving, 2 years post-treatment. 5/8 participants remained abstinent for more than 3 years. Moreover, improved quality of life and alleviated mental disorders were observed.

Bold values have been used to highlight the outcome of interest and the brain region targeted, to improve clarity. Substance use disorder investigated is also shown in bold.

**Table 4:** A Summary of End-of-Treatment Substance-use Outcomes in Neuromodulation for Substance Use Disorder Studies. [*N* = 4036, Participants; 94 Studies].

Substance Use Disorder	Neuromodulation Method					
	Repetitive Transcranial Magnetic Stimulation (rTMS) [Total *N* = 2406; 51 Studies]		Transcranial Direct Current Stimulation (tDCS) [Total *N* = 1582; 36 Studies]		Deep Brain Stimulation (DBS) [Total *N* = 48; 7 Studies]	
	Population	Studies with Positive Outcomes *(Effect Size – Active vs. Control)*	Population	Studies with Positive Outcomes *(Effect Size – Active vs. Control)*	Population	Studies with Positive Outcome *(Effect Size – Post vs. Pre.)*
**Alcohol** [*N* = 1369; 34 Studies]	*n* = 607 (16 Studies)	7/16 (**44%**) *Hedge’s g* = *−1.01, 95% CI [−1.62, −0.40]*	*n* = 734 (14 Studies)	9/14 (**64%**) *Hedge’s g* = *−0.31, 95% CI [−0.62, 0.002]*	*n* = 28 (4 Studies)	4/4 (**100%**) *Hedge’s g* = *−2.36, 95% CI [−3.31, −1.41]*
**Tobacco** [*N* = 1239; 28 Studies]	*n* = 781 (16 Studies)	14/16 (**88%**) *Hedge’s g* = *−1.36, 95% CI [−2.09, −0.63]*	*n* = 448 (11 Studies)	7/11 (**64%**) *Hedge’s g* = *−0.50, 95% CI [−0.87, −0.13]*	*n* = 10 (1 Study)	1/1 (**100%**) *Hedge’s g* = *−0.40, 95% CI [−1.28–0.49]*
**Cannabis** [*N* = 33; 2 Studies]	*n* = 33 (2 Studies)	1/2 (**50%**) *Hedge’s g* = *0.04, 95% CI [−0.49, 0.57]*	*n* = 0 (0 Studies)	NA	*n* = 0 (0 Studies)	NA
**Cocaine** [*N* = 321; 9 Studies]	*n* = 227 (6 Studies)	3/6 (**50%**) *Hedge’s g* = *−0.73, 95% CI [−1.57, 0.11]*	*n* = 94 (3 Studies)	1/3 (**33%**) *Hedge’s g* = *−0.19, 95% CI [−0.27, −0.11]*	*n* = 0 (0 Studies)	NA
**Methamphetamine** [*N* = 714; 13 Studies]	*n* = 519 (8 Studies)	7/8 (**88%**) *Hedge’s g* = *−1.45, 95% CI [−3.22, 0.32]*	*n* = 195 (5 Studies)	5/5 (**100%**) *Hedge’s g* = *−0.33, 95% CI [−0.89, 0.23]*	*n* = 0 (0 Studies)	NA
**Opioid** [*N* = 360; 9 Studies]	*n* = 239 (4 Studies)	3/4 (**75%**) *Hedge’s g* = *−0.99, 95% CI [−2.25, 0.27]*	*n* = 111 (3 Studies)	3/3 (**100%**) *Hedge’s g* = *−1.85, 95% CI [−2.47, −1.23]*	*n* = 10 (2 Studies)	2/2 (**100%**) *Hedge’s g* = *−5.75, 95% CI [−7.97 to −3.53]*

Bold values have been used to highlight the percentage of studies with positive outcomes, as well as the substance use disorder investigated, for improved clarity as well.

The Cochrane Risk-of-Bias Tool (RoB-2) [51] assessed quality of included RCTs. Studies with a high risk of bias were subsequently excluded if at least four domains were considered of moderate risk, or if two or more domains were flagged as high risk. The Risk Of Bias In Non-randomized Studies of Interventions (ROBINS-I) [52] tool assessed risk of bias in non-randomized studies (DBS Studies); all extracted DBS studies were included in this review.

### Meta-analysis

To quantify NM effects, we performed meta-analyses on rTMS and tDCS studies investigating alcohol and tobacco use disorders. Acute versus repeated stimulation were independently evaluated. Meta-analyses were conducted when three or more studies evaluated a synonymous outcome (craving, cue-induced craving, and/or consumption).

We utilized standardized mean difference (SMD; Hedge’s *g*) with 95% confidence intervals (CI’s) in each selected meta-analysis to calculate the effect size of NM-related changes in alcohol and tobacco craving, cue-induced craving, and/or consumption (*p* ≤ 0.05, two-tailed). Random-effects models pooled individual SMDs, and used data from studies that reported end-of-treatment substance use data from active and control treatment arms. Negative values indicated that active stimulation produced greater reductions in craving, cue-induced craving, and/or consumption compared to sham treatment. The I^2^ statistic estimated between-trial heterogeneity; I^2^ of ≤40% was considered low heterogeneity, 40–60% moderate heterogeneity, and >60% high heterogeneity [53]. Meta-analyses were performed using R version 4.3.1 [54] with package metafor [55].

## Results

We identified a total of 94 studies that met our inclusion criteria, with a total of 4306 participants.

### Repetitive transcranial magnetic stimulation (rTMS)

Fifty-one studies investigating rTMS as treatment for SUDs were identified, with 2406 participants receiving either active or control treatment (sham stimulation or no treatment; Table 1).

#### Alcohol

Sixteen studies [56–71] investigated the effects of rTMS for alcohol use disorder (AUD). Eleven studies used multiple active sessions (10–20 sessions) with HF stimulation (10–20 Hz) targeting right, left, or bilateral dorsolateral prefrontal cortex (DLPFC), medial prefrontal cortex (mPFC) or insula [61–71]. Findings were mixed, with seven studies [61, 63–65, 68, 71] demonstrating significant post-TMS reductions in alcohol craving and/or consumption compared to sham stimulation. Notably, 3/7 positive studies applied deep TMS using various H-coils as opposed to the traditional Figure-8 coil, suggesting that this technology may be particularly efficacious in treating AUD. Two studies employed the H-1 coil to target the mPFC and bilateral DLPFC, respectively, whilst one opted for the H-7 coil to target both the mPFC and anterior cingulate cortex (ACC) concurrently. One study [71] applied a 10 session cTBS stimulation protocol to the mPFC, with significant reductions in alcohol craving.

**Fig. 2:**
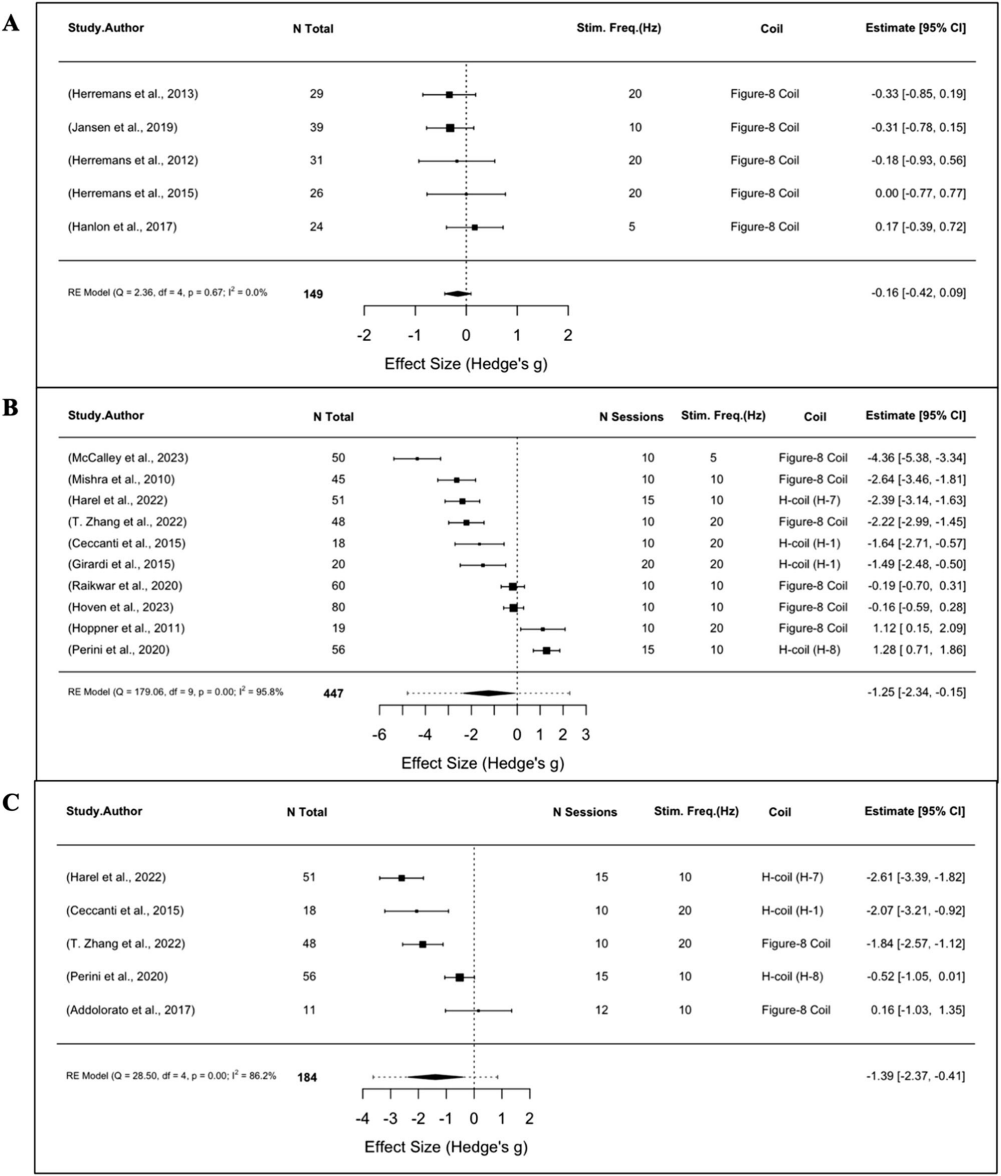
Meta-analyses of AUD studies using rTMS. Forest plots of studies evaluating (**A**) alcohol craving following a single-session of rTMS (**B**) alcohol craving following multi-session rTMS (**C**) alcohol consumption following multi-session rTMS.

Of these eleven studies, ten were combined in a meta-analysis to determine the effects of repeated rTMS stimulation on alcohol craving (*n* = 447). Active rTMS significantly reduced craving scores in AUD compared to sham (SMD = −1.25, 95% CI: −2.34 to −0.15, *p* = 0.02, I^2^ = 95.8%; Fig. 2B). Similarly, meta-analysis of five repeated rTMS trials (*n* = 184) demonstrated that multiple rTMS sessions produced greater reductions in alcohol consumption than sham (SMD = −1.39, 95% CI: −2.37 to −0.41, *p* < 0.01, I^2^ = 86.2%; Fig. 2C).

Five studies [56–60] evaluated the effects of a single active (10–20 Hz) stimulation session and found no significant improvements in alcohol craving or consumption post-TMS when compared to sham. Four studies [56–58, 60] targeted the right DLPFC, while one [59] targeted the left frontal pole with cTBS. Accordingly, meta-analysis of craving outcomes in these five trials (*n* = 149) revealed that acute active versus sham rTMS did not significantly decrease craving (SMD = −0.16, 95% CI: −0.42 to 0.09, *p* = 0.21, I^2^ = 0%; Fig. 2A).

#### Tobacco

Sixteen studies [37, 72–86] examined efficacy of rTMS for tobacco use disorder (TUD). All studies demonstrated reductions in tobacco craving/cue-induced craving and/or cigarette consumption following active versus sham rTMS, with the exception of Li et al. [72] and Kozak et al. [73]. While Li et al. applied a single 10 Hz stimulation session targeting the left DLPFC, Kozak et al. [73] tested multiple HF sessions (20 Hz) targeting the bilateral DLPFC in individuals with comorbid schizophrenia (SCZ). However, Moeller et al. [85] applied deep-TMS to the PFC and insula using the H-4 coil in nicotine-dependent SCZ patients and found that active stimulation increased the latency to smoke, suggesting reduced motivation. Similarly, Ibrahim and colleagues [86] applied multiple sessions of active versus sham deep TMS to insular cortex in smokers receiving concurrent varenicline treatment, and found significant rTMS-related effects in smoking abstinence at Week 12.

Dinur-Klein et al. [84] and Zangen et al. [37] also applied deep-TMS to the lateral PFC and insula using the H-ADD and H-4 coils respectively, and found significant reductions in tobacco consumption and craving [37, 84]. Importantly, Dinur-Klein et al. [84] applied both 1 Hz (LF) and 10 Hz (HF) stimulation to the lateral prefrontal cortex (PFC) and insula, finding that cigarette consumption decreased significantly only in the 10 Hz condition. These studies were amongst the largest studies of NM for SUDs, with sample sizes of 115 and 262 respectively. The study by Zangen et al. [37] is the only multisite clinical trial in the addiction NM field, and led to FDA clearance of the H-4 coil for smoking cessation.

Notably, while Trojak et al. [81] reported positive results, findings were not maintained at follow-up (12 weeks), signifying a lack of durability in long-term outcomes, though this was the only study to apply LF stimulation (1 Hz) exclusively.

Additionally, two studies [77, 84] investigated cue-induced provocation prior to stimulation, and found that presentation of smoking cues reduced cigarette consumption and cue-induced craving, respectively.

**Fig. 3:**
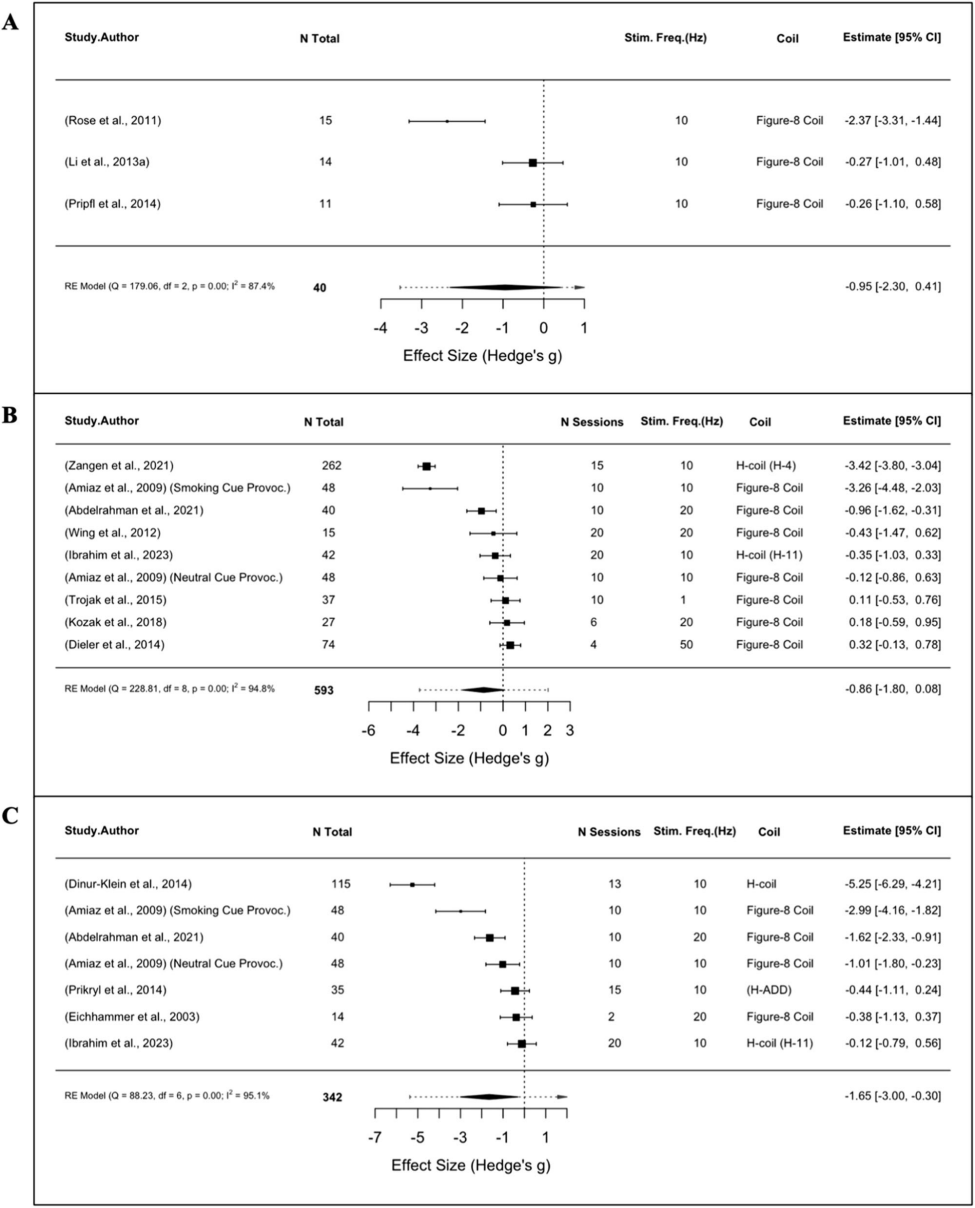
Meta-analyses of TUD studies using rTMS. Forest plots of studies evaluating (**A**) tobacco cue-induced craving following a single-session of rTMS (**B**) tobacco craving following multi-session rTMS (**C**) tobacco consumption following multi-session rTMS.

Meta-analyses were performed on acute and repeated rTMS for TUD. Of four single-session rTMS studies, three reported cue-induced craving (*n* = 40) and were subsequently evaluated, indicating no significant effect of a single active versus sham stimulation session (SMD = −0.95, 95% CI: −2.30 to 0.41, *p* = 0.17, I^2^ = 87.4%; Fig. 3A). Of twelve multi-session studies, six reported tobacco consumption (*n* = 342) and eight reported subjective craving (*n* = 593). While repeated rTMS significantly reduced cigarette use (SMD = −1.65, 95% CI: −3.00 to −0.30, *p* = 0.01, I^2^ = 95.1%; Fig. 3C), there was no significant effect of active versus sham stimulation on craving (SMD = −0.86, 95% CI: −1.80 to 0.08, *p* = 0.07, I^2^ = 94.8%; Fig. 3B).

#### Cannabis

Only two RCTs [87, 88] examined the use of rTMS for cannabis use disorder (CUD). Sahlem et al. [87] used a randomized, sham-controlled, crossover design to investigate therapeutic effects of a single 10 Hz stimulation session applied to left DLPFC, finding no significant differences in cannabis craving compared to sham. Kozak-Bidzinski et al. [88] applied 20 sessions of 20 Hz rTMS to bilateral DLPFC using a parallel groups design in participants with CUD and schizophrenia. Non-significant reductions in cannabis consumption were noted post-TMS versus sham (60 versus 5%), and trends towards reductions in urine toxicology (carboxy-tetrahydrocannabinol) and craving were observed.

#### Cocaine

Six studies [59, 89–93] investigated rTMS for cocaine use disorder. Two studies demonstrated a significant decrease in cocaine craving following multiple sessions of 15 Hz rTMS to the left DLPFC. Martinez et al. [91] applied both 1 Hz and 10 Hz stimulation to mPFC and ACC using the H-7 coil, finding no significant effect on cocaine craving, though a reduction in cocaine self-administration was present in the 10 Hz condition versus 1 Hz rTMS and sham. Conversely, Bolloni et al. [89] found no significant effects of deep TMS on cocaine consumption when targeting the PFC with H-1 coil, though there was a trend for decreased consumption between baseline and 6-months post-TMS in the active group. Hanlon et al. [59] applied a single stimulation session, finding no treatment-related effects on craving following cTBS to the left frontal pole.

#### Methamphetamine

Eight studies [94–101] investigated the use of rTMS for methamphetamine (MA) use disorder. Seven studies [95–101] exhibited significant improvements in MA unconditioned and cue-induced craving and/or consumption following multiple active rTMS sessions (5–20) targeting the left DLPFC or left PFC (1–10 Hz), compared to sham treatment. Interestingly, Li et al. [94] found that a single 1 Hz stimulation session applied to the left DLPFC increased cue-induced MA craving compared to sham. Notably, three studies [98–100] adopted iTBS and/or cTBS stimulation parameters and reported positive results consistent with standard rTMS.

#### Opioids

Four studies [102–105] evaluated outcomes in opioid use disorder (OUD) patients following multiple HF rTMS sessions (5–40) targeting the left DLPFC. Three studies [103–105] reported significant improvements in opioid craving and/or cue-induced craving, with the exception of Tsai et al. [102] who evaluated treatment effects in participants receiving concurrent methadone maintenance therapy. Although there was no significant impact on opioid craving or consumption, an improvement in depressive symptoms was present post-treatment. Li et al. [105] also observed improvements in depressive symptoms, in conjunction with reduced opioid craving, though their participants received concurrent occupational therapy. Liu et al. [104] applied both 1 Hz and 10 Hz stimulation to the left DLPFC, finding that both conditions produced similar reductions in cue-induced opioid craving compared to no treatment.

### Transcranial direct current stimulation (tDCS)

Thirty-six studies investigating tDCS as treatment for SUDs, with 1582 participants receiving either active or control treatment (sham stimulation or no treatment; Table 2).

#### Alcohol

Fourteen studies [106–119] examined the effects of tDCS for AUD. Nine [106, 108–110, 113, 114, 116, 117, 119] demonstrated positive effects on alcohol craving and/or consumption following right or left anodal tDCS to DLPFC. While single stimulation sessions of right anodal and left anodal tDCS to the DLPFC demonstrated comparable effects, multi-session studies showed that right anodal DLPFC stimulation was consistently effective [113, 114, 119] but left anodal DLPFC stimulation was inconsistent [108, 110–112]. Variations of stimulation intensity (1–2 mA) and duration (10–30 min) were explored, though these differences did not produce consistent outcomes.

**Fig. 4:**
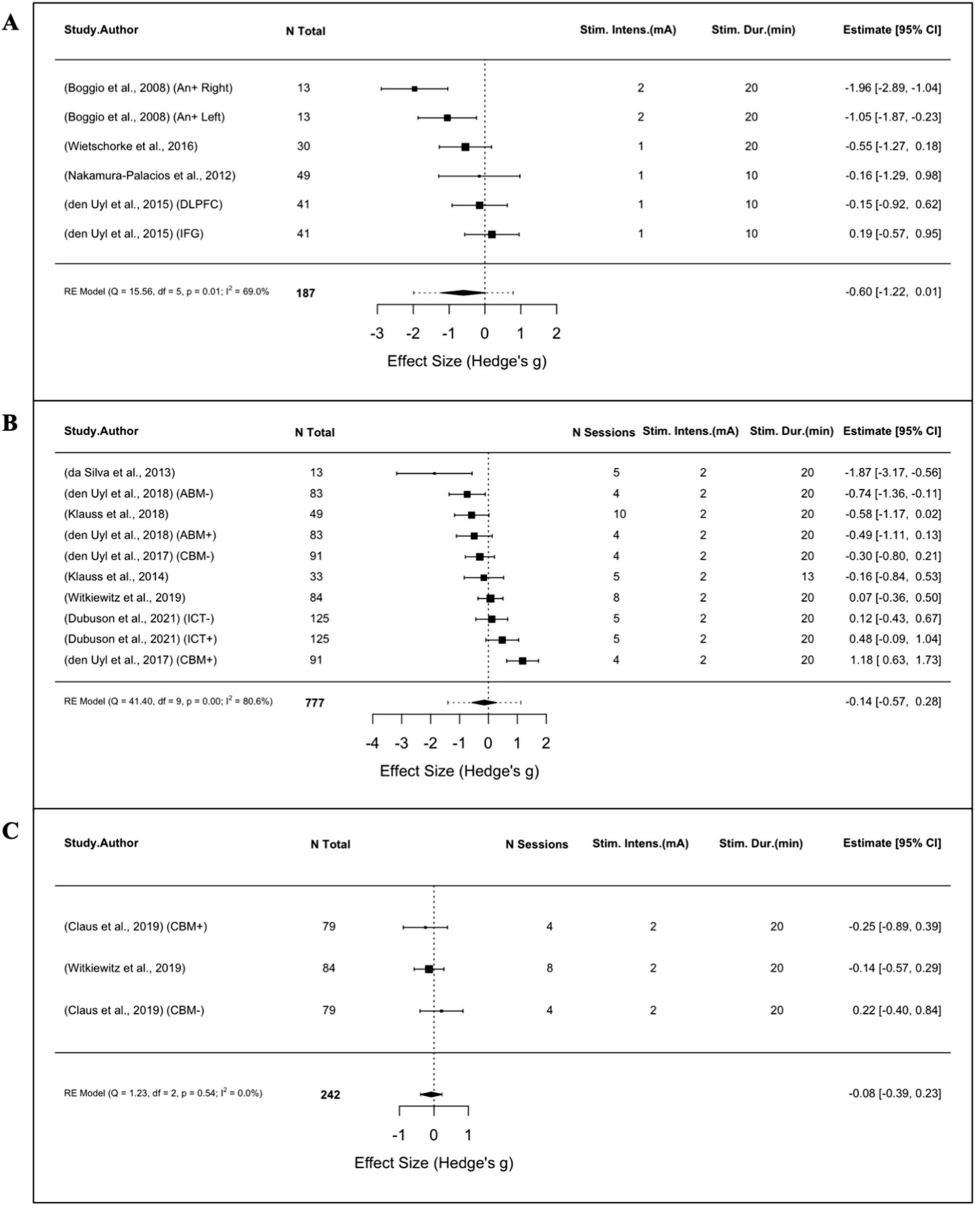
Meta-analyses of AUD studies using tDCS. Forest plots of studies evaluating (**A**) alcohol craving following a single-session of tDCS (**B**) alcohol craving following multi-session tDCS (**C**) alcohol consumption following multi-session tDCS.

While nine studies reported positive effects on alcohol use outcomes following active tDCS, meta-analyses of craving and consumption outcomes in single- and multi-session studies did not reveal significant SMDs for active versus sham stimulation. Analysis of subjective craving from four single-session trials (*n* = 187) were non-significant (SMD = −0.60, 95% CI: −1.22 to 0.01, *p* = 0.06, I^2^ = 69.0%; Fig. 4A), as were sub-group analyses of craving (*n* = 777, SMD = −0.14, 95% CI: −0.57 to 0.28, *p* = 0.51, I^2^ = 80.6%; Fig. 4B) and consumption (*n* = 242, SMD = −0.08, 95% CI: −0.39 to 0.23, *p* = 0.62, I^2^ = 0%; Fig. 4C) from eight multi-session trials.

#### Tobacco

Eleven studies [120–130] were conducted on tDCS in TUD. All studies applied 2.0 mA stimulation for 15–30 min, except for Falcone et al. [123] and Meng et al. [121] both of whom applied 1.0 mA stimulation for 20 min. Seven studies, including Falcone et al. and Meng et al. reported positive effects on tobacco craving and/or cigarette consumption [121, 123–127, 129], with right anodal DLPFC stimulation being most effective, particularly with multi-session protocols [125–129]. Notably, Ghorbani-Behnam et al. [129] compared extended tDCS treatment (20 sessions over 12 weeks) with a shorter treatment duration (20 sessions over 4 weeks), with 8 weeks of bupropion and sham stimulation. Results showed that longer durations of tDCS resulted in the highest abstinence rate at 6 months post-treatment (25.7%).

**Fig. 5:**
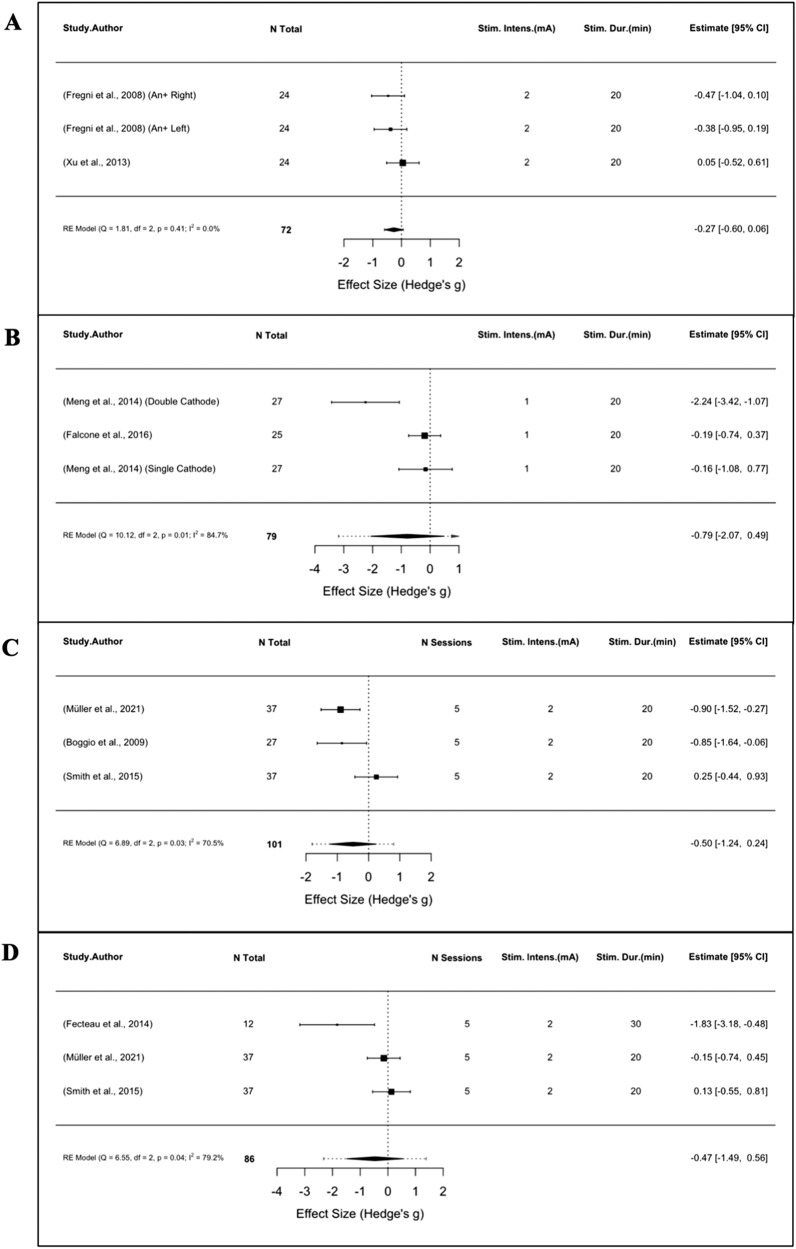
Meta-analyses of TUD studies using tDCS. Forest plots of studies evaluating (**A**) tobacco craving following a single-session of tDCS (**B**) tobacco consumption following a single-session of tDCS (**C**) tobacco craving following multi-session tDCS (**D**) tobacco consumption following multi-session tDCS.

While seven studies reported independent improvements in tobacco-related outcomes, meta-analysis did not reflect similar effects. From four single-session studies, sub-group analyses of craving (*n* = 72, SMD = −0.27, 95% CI: −0.60 to 0.06, *p* = 0.11, I^2^ = 0%; Fig. 5A) and consumption (*n* = 79, SMD = −0.79, 95% CI: −2.07 to 0.49, *p* = 0.22, I^2^ = 84.7%; Fig. 5B) did not produce significant effects with active versus sham stimulation. Similarly, in four multi-session trials, subgroup analyses of craving (*n* = 101, SMD = −0.50, 95% CI: −1.24 to 0.24, *p* = 0.19, I^2^ = 70.5%; Fig. 5C) and consumption (*n* = 86, SMD = −0.47, 95% CI: −1.49 to 0.56, *p* = 0.37, I^2^ = 79.2%; Fig. 5D) were non-significant.

#### Cocaine

Three studies [131–133] examined tDCS on cocaine craving using right anodal DLPFC stimulation, reporting conflicting results. While Batista et al. [131]. observed a reduction in cocaine craving after 5 sessions of 2 mA/20 min tDCS, Verveer et al. [132]. and Gaudreault et al. [133]. found no significant effects on craving following 10 active 2 mA/13 min or 15 active 2 mA/20 min sessions, respectively.

#### Methamphetamine

Five studies [134–138] investigated the effects of tDCS on MA use disorder, all of which reported a significant reduction in MA unconditioned or cue-induced craving compared to sham following right anodal DLPFC tDCS. Four studies [134–137] applied 2.0 mA stimulation for 20 min, whereas Xu et al. [138] combined 1.5 mA tDCS with computerized cognitive addiction therapy (CCAT). While both studies by Shahbabaie et al. [134, 135] examined effects of a single stimulation session, the remaining three studies [136–138] opted for a multi-session protocol (5–20 sessions). Notably, 4/5 [134–137] of these studies evaluated males only, while the remaining study examined only female participants [138].

#### Opioids

Three studies [139–141] were conducted on tDCS treatment efficacy for OUD. Two studies [139, 140] applied ten sessions of 2.0 mA tDCS to the DLPFC for 20 min. Taremian et al. [140] evaluated opioid craving and depressive symptoms in participants receiving methadone, and compared right anodal DLPFC stimulation with sham. Active tDCS significantly reduced opioid craving and depressive symptoms, compared to sham, and methadone alone. Eskandari et al. [139] compared left anodal DLPFC stimulation with right anodal DLPFC stimulation and sham, observing a significant reduction in craving in all groups; active groups exhibited greater effects. Wang et al. [141] applied a single stimulation session targeting the fronto-parietal-temporal area at 1.5 mA for 20 min. Despite these differences, a significant decline in heroin craving was observed, which persisted with the presentation of opioid-related cues.

### Deep brain stimulation (DBS)

Seven studies investigated DBS as SUD treatment, with 48 participants receiving active or sham stimulation (Table 3).

#### Alcohol

Four studies [142–145] investigated effects of DBS on AUD by targeting the NAc. All studies observed significant decreases in alcohol consumption and/or craving post-treatment. Notably, Bach et al. [145] (*N* = 12) was the first to compare active and sham DBS and found significant improvements in substance use and craving following 6-months of active stimulation.

#### Tobacco

One study examined the use of DBS on TUD by targeting the NAc. Kuhn et al. [146] found that 3/10 TUD participants in their study quit smoking post-treatment, while the remaining seven participants showed a significant decline in tobacco craving and cigarette consumption.

#### Opioids

Two studies [147, 148] examined effects of DBS treatment in heroin-dependent participants and reported significant reductions in opioid craving and an increase in opioid abstinence. While Kuhn et al. [147] targeted the NAc exclusively, Chen et al. [148] applied simultaneous stimulation to anterior limb of the internal capsule (ALIC) and NAc.

## Discussion

We systematically reviewed the cumulative literature on the efficacy of NM (rTMS, tDCS, DBS) for SUD treatment (Table 4). Findings were inconsistent across each stimulation methodology, and varied significantly with respect to SUD. This may be attributed to variations in treatment parameters, symptom severity across SUD participants, use of adjunctive treatment interventions and population heterogeneity, including the presence of comorbid psychiatric disorders, age, sex, and treatment history.

Nonetheless, findings from rTMS and tDCS studies demonstrated several commonalities. For rTMS, positive outcomes when treating tobacco, stimulant and opioid use disorders were observed, as indicated by post-treatment reductions in subjective and cue-induced substance craving and/or consumption when compared to sham treatment. Accordingly, effect sizes were clinically relevant (Hedge’s *g* > 0.5) but highly variable, consistent with heterogeneity of the published literature [9]. Furthermore, meta-analyses found that multi-session active versus sham rTMS was particularly effective in reducing tobacco consumption, but effects on tobacco craving were non-significant. Interestingly, effects of rTMS on AUD were less consistent, with 7/16 studies demonstrating significant improvements. Subsequent meta-analyses found that multi-session rTMS produced significantly greater reductions in alcohol craving and consumption. tDCS studies were promising in the treatment of tobacco, alcohol, stimulant, and opioid use disorders, as suggested by medium effect sizes (Table 2). However, meta-analyses of tDCS trials for AUD and TUD found that both single- and multi-session stimulation were not superior to sham stimulation in reducing craving or consumption, suggesting that rTMS may be superior to tDCS for these SUDs.

DBS produced reductions in craving, consumption and/or abstinence in alcohol, tobacco, and opioid use disorders. Available data is limited to case-series making it difficult to calculate effect sizes (Table 3), with the exception of one randomized sham-controlled study in AUD [145]. Sample sizes in DBS studies were low (ranging 2–12, averaging 6.9 ± 3.1 participants), suggesting the need for larger samples and randomized controlled trials.

### Treatment parameters

Variability in treatment efficacy across NM studies may be attributed to differences in stimulation parameters (e.g., stimulation target, frequency, intensity, treatment duration and sample size/demographics). For both rTMS and tDCS studies, multi-session protocols are more effective than single-sessions protocols, as indicated by larger effect sizes and the number of positive outcome studies (see Tables 1–4). This is consistent with previous reports in the addictions neuromodulation literature [149]. However, total number of sessions needed to produce long-lasting effects is unclear and requires further investigation. For rTMS, the most commonly used paradigm across substances was 10–20 sessions once daily. In contrast, studies investigating TMS in depression suggest ≥30 sessions are needed for treatment durability [150]. While studies demonstrated persistent effects, including post-TMS reductions in 3-month alcohol [71] and cigarette consumption [82] after only 10 sessions of rTMS, durability of these effects remains uncertain as there is lack of long-term follow-up and biochemical verification beyond 1-month. Amiaz et al. [77] found that reductions in cigarette consumption after 10 sessions of rTMS were not maintained at 6-months. Similarly, number of tDCS sessions needed remains unclear due to lack of long-term follow-up. tDCS protocols were also considerably shorter, with all but two studies [129, 138] applying ≤10 sessions overall. Interestingly, Ghorbani Behnam et al. [129] applied 20 total sessions and found that when these sessions were distributed over a longer period of time (12 versus 4 weeks), tobacco abstinence was considerably higher at 6-month follow-up. Accordingly, session frequency may also play an important role. Moreover, potential effects of an accelerated stimulation paradigm (e.g. more than one session daily) should also be further investigated. Studies in depression have found that accelerated protocols are safe and well-tolerated, and perform comparably to standard once-daily rTMS [151–153]. Martinotti et al. [93] conducted the only randomized sham-controlled addictions study to adopt such an accelerated stimulation approach, but reported unfavourable cocaine use outcomes following twice daily stimulation. Nonetheless, Steele and colleagues [154] have found that three iTBS sessions/day for 10 days was tolerable and reduced cocaine consumption.

The need for maintenance sessions following initial stimulation treatment should be further evaluated to increase durability [155]. Two studies incorporated weekly reminder sessions following 15 daily HF deep-TMS sessions, and found that reductions in alcohol consumption [68] and tobacco craving [37] persisted 3-months post-treatment. However, Amiaz et al. [77] found that improvements in tobacco use outcomes following 10 HF rTMS sessions and 8 maintenance sessions did not persist at 6-months; this may reflect the effects of the coil (Figure-8 vs. H-coil) or the number of initial sessions (10 versus 15).

Four rTMS studies [83, 84, 91, 104] compared the effects of LF (1 Hz) and HF (10 Hz) stimulation and found that 10 Hz rTMS significantly reduced substance craving and/or consumption, suggesting that HF rTMS stimulation parameters have greater therapeutic potential in comparison to LF stimulation. Accordingly, most rTMS studies used HF stimulation (e.g., ≥5 Hz) regardless of SUD. For tDCS studies, the effects of stimulation intensity (1 mA vs. 2 mA) were less clear. However, tDCS outcomes were more promising when stimulation sessions were of longer duration (>15 min).

Cue-exposure prior to rTMS may activate craving-related neurocircuitry, and subsequent stimulation could then disrupt drug-related memory consolidation [156]. Accordingly, Dinur-Klein et al. [84] incorporated smoking cue exposure prior to HF deep TMS and found that it reduced cigarette consumption. Amiaz et al. [77] evaluated differential effects of both neutral and smoking cues prior to HF rTMS, finding that smoking cues reduced cue-induced tobacco craving. This expands on previous findings in both PTSD [157] and OCD [158], wherein provocation using brief cue exposure prior to treatment alleviated symptoms compared to no cue provocation. Future studies should determine whether cue exposure should be utilized in all rTMS and tDCS protocols.

There were inconsistencies for rTMS in AUD treatment, with positive outcomes reported in 44% of studies. Nonetheless, deep TMS was effective when compared to rTMS using a Figure-8 coil, suggesting that the H-coil may be advantageous when treating AUD due to targeting of deep brain structures (e.g., insula, nucleus accumbens). Subsequent meta-analyses did find positive effects of multi-session rTMS on alcohol craving and consumption. However, given that there are several evidence-based treatments available for AUD [159], we suggest that neuromodulation treatment development should be focused on SUDs with a lack of evidence-based biological treatments, such as cannabis and stimulants.

### Target brain region

Substance use outcomes with NM are influenced by targeted brain region, as well as the subsequent bilateral or unilateral stimulation of regions of interest. Most rTMS studies for SUDs have targeted the DLPFC (38/50 studies). rTMS targeting the left DLPFC produced predominantly positive effects and clinically relevant effect sizes when treating tobacco, stimulant and opioid use disorders, while those stimulating the right or bilateral DLPFC were less effective (Table 1). In contrast, studies in AUD were not responsive to left DLPFC rTMS, though right and bilateral DLPFC stimulation was effective when multiple sessions were conducted. Alternative regions were less commonly studied. Notably, the mPFC/frontal pole (with or without concurrent stimulation of ACC) emerged as a novel therapeutic target, particularly with a deep TMS protocol with H-coil technology, as indicated by studies with alcohol [63, 68] and cocaine [91]. Targeting bilateral PFC and insular cortex with deep TMS may also be effective in alcohol and tobacco treatment [37, 66, 84, 86].

Both DLPFC and mPFC have emerged as leading rTMS targets; much remains unknown about the mechanism by which rTMS induces its therapeutic effects in SUDs. An understanding of rTMS-induced alterations in SUD-related brain circuitry is limited as very few studies have incorporated neuroimaging. Furthermore, there is much uncertainty surrounding optimal target locations, both for specific SUDs and individual patients, as there have been no direct head-to-head comparisons of different active rTMS targets. Consequently, it is possible that alternate targets may be required for distinct SUDs. Interestingly, there is evidence that the Default Mode Network may be a SCZ-specific network of tobacco dependence [160]. It is critical that rTMS clinical trials include brain-based measures (e.g., MRI, EEG) in order to elucidate mechanisms of action and identify optimal treatment targets.

With respect to tDCS, right anodal DLPFC stimulation appears to be most efficacious across all substances. However, right anodal DLPFC studies had considerably more stimulation sessions (≥5 sessions) than those applying left anodal DLPFC (≤5 sessions) stimulation. Thus, observed differences may be related to treatment duration, and future studies should explore longer durations of left anodal DLPFC tDCS.

Importantly, stimulation sites for rTMS and tDCS are conventionally identified using the 10–20 EEG system or by measuring distances from predefined external landmarks. While this one-size-fits-all approach produces approximate targeting of specified regions, it does not consider inter-individual differences in brain morphology and network architecture. Neuronavigation-guided NM with magnetic resonance imaging (MRI) may achieve greater precision with personalized targets. rTMS studies in depression have demonstrated the benefits of such an approach and found that clinical outcomes were significantly improved when patients were stimulated closer to fMRI-personalized targets [161]. Selected rTMS studies integrated MRI-neuronavigation [56–58, 60, 75, 81, 90], though the number of studies was insufficient to distinguish its effectiveness in comparison to non-personalized targeting. No tDCS studies were present. Consequently, future randomized control trials are warranted to assess the clinical potential of neuronavigation-guided personalized rTMS and tDCS. Most DBS studies targeted the NAc, and were consistently positive.

### Alternate neuromodulation modalities

Other NM methods that are less frequently used and excluded from this review include Electroconvulsive Therapy (ECT) [162], Magnetic Seizure Therapy (MST) and Transcranial Alternating Current Stimulation (tACS) [163]. Studies examining their effects on SUDs are limited. We also excluded invasive ACC stimulation; ACC implants have shown positive effects, particularly for AUD, although adverse events have been reported [164].

### Psychiatric comorbidities

Only a few studies have tested neuromodulation interventions in populations with comorbid psychiatric disorders. Notably, 3/4 of rTMS studies that examined TUD participants with co-occurring SCZ observed significant reductions in tobacco craving and consumption [78, 80, 85] (Table 1). Prevalence of tobacco use in SCZ is 60–80% and contributes to a 25-year decreased life expectancy in SCZ [165], emphasizing the therapeutic potential of rTMS for this comorbidity. Moreover, SCZ patients have high rates of cannabis misuse [166]. Kozak-Bidzinski et al. [88] studied rTMS in outpatients with SCZ and CUD (*N* = 19). Although the difference in cannabis use was not statistically significant, larger reductions (~60%) were observed in the active (*n* = 9) versus sham (*n* = 10) group, highlighting its treatment potential. Ultimately, these NM methods show promise in treating co-occurring SUD and psychiatric disorders, warranting further research in clinical trials with larger sample sizes.

### Strengths and limitations

This comprehensive systematic review and meta-analysis contributes substantially to the literature on NM for SUDs for the following reasons: (1) We calculated effect sizes for each study across all three stimulation modalities, and where applicable, conducted a meta-analysis of the published data, to compare and contrast these treatment outcomes. This is the first comprehensive systematic review of the addiction NM literature to include meta-analytic comparisons; (2) We evaluated the treatment efficacy of each stimulation technique, with respect to each SUD and the stimulation parameters applied, to identify their differential effects across substances; (3) We included several new studies that have been published since the reviews by Salling and Martinez [8] and Coles and colleagues [9].

However, there were some limitations. First, there was significant variability in the number of studies for each SUD and NM methodology. Many of these studies were also preliminary (sample size <40 participants). Second, studies were not balanced for sex, with an emphasis on males. Thus, sex-related differences in treatment outcomes are unclear. Third, there was variability in outcomes evaluated (e.g., craving vs. consumption) and in methods used to measure them (e.g., biochemical verification versus self-report). Fourth, as substance use was the primary outcome of interest, associated outcomes such as psychiatric symptoms and cognition were secondary and not always reported. Finally, treatment effects were quantitively assessed using end-of-treatment data due to heterogeneity in follow-up periods. Thus, enduring effects of NM interventions cannot be adequately determined.

### Conclusions and future directions

There is considerable promise for the use of NM therapies in SUDs. Nonetheless, further research is required to determine clinical safety and efficacy. Future studies should focus on optimizing stimulation parameters and regimens for these NM methods, with emphasis on stimulation duration, number of treatment sessions needed to produce enduring effects, accelerated treatment paradigms, stimulation frequency and intensity and targeted brain region. Assessment of enduring effects of NM treatment using biochemical verification at extended time-points and the need for maintenance sessions following treatment cessation to optimize clinical outcomes should be emphasized. Neuroimaging data (fMRI) should be acquired prior to, during, and following treatment to elucidate the underlying neural mechanisms mediating treatment effects. Moreover, MRI-neuronavigation may address potential discordance between coil/electrode placement and region of interest, potentially improving treatment efficacy.

Finally, greater emphasis on co-occurring psychiatric disorders is needed. rTMS may be a promising intervention for patients with SCZ and concurrent SUDs, warranting larger randomized sham-controlled trials. Finally, the potential of adjunctive psychotherapeutic and/or pharmacological intervention should be determined, which may improve substance use outcomes [81]. While some studies have implemented concurrent pharmacological interventions [78], few have parsed the clinical impact of each therapy for augmentation of NM outcomes.

### Supplementary information

Supplemental Material
