# Supplementary material for: A systematic review and meta-analysis of neuromodulation therapies for substance use disorders
Source: Neuropsychopharmacology. 2023 Dec 12;49(4):649–80. doi: 10.1038/s41386-023-01776-0 (PMC10876556; doi:10.1038/s41386-023-01776-0)
Supplement: Supplementary file 1 — Supplemental Material [file 41386_2023_1776_MOESM1_ESM.docx]

**Supplementary Table 1.** Search strategy: key words, phrases, and search terms

| **Medline, PubMed**  *(Medical Subject Headings (MeSH) terms)* | **PsycINFO**  *(Thesaurus)* |
| --- | --- |
| ***Concept 1: Substance Use Disorder*** | |
| “Substance-Related Disorders” | “Substance Related and Addictive Disorders” |
| ***Concept 2: Repetitive Transcranial Magnetic Stimulation*** | |
| “Transcranial Magnetic Stimulation” OR “TMS” | “Transcranial Magnetic Stimulation” |
| ***Concept 3: Transcranial Direct Current Stimulation*** | |
| “Transcranial Direct Current Stimulation” OR “tDCS” | “Transcranial Direct Current Stimulation” |
| ***Concept 4: Deep Brain Stimulation*** | |
| “Deep Brain Stimulation” OR “DBS” | “Deep Brain Stimulation” |

1197 studies identified through database (OVID Medline, PsychINFO, PubMed) searching

94 studies included in systematic review

146 full-text studies assessed for eligibility

579 records excluded

472 duplicate records removed by Covidence

725 title and abstracts screened

52 records excluded:

- 23 Wrong outcomes
- 11 No sham/control condition
- 4 Wrong patient population
- 3 Wrong intervention
- 3 No diagnosis of SUD with standardized criteria
- 3 Not a primary research article
- 3 Secondary analysis of included paper
- 2 Not accessible

**Supplementary Figure 1:** PRISM-A Flow Diagram for Study Selection
